# Distribution and accumulation of dietary ergothioneine and its metabolites in mouse tissues

**DOI:** 10.1038/s41598-018-20021-z

**Published:** 2018-01-25

**Authors:** Richard Ming Yi Tang, Irwin Kee-Mun Cheah, Terry Shze Keong Yew, Barry Halliwell

**Affiliations:** 1 National University of Singapore Graduate School for Integrative Sciences and Engineering, Singapore, Singapore; 2 Department of Biochemistry, Yong Loo Lin School of Medicine, National University of Singapore, 28 Medical Drive, Singapore, Singapore

## Abstract

L-ergothioneine (ET) is a diet-derived amino acid that accumulates at high concentrations in animals and humans. Numerous studies have highlighted its antioxidant abilities *in vitro*, and possible cytoprotective capabilities *in vivo*. We investigated the uptake and distribution of ET in various organs by a highly sensitive and specific liquid chromatography coupled tandem mass spectrometry (LC-MS/MS) technique, both before and after oral administration of pure ET (35 and 70 mg/kg/day for 1, 7, and 28 days) to male C57BL6J mice. ET primarily concentrates in the liver and whole blood, and also in spleen, kidney, lung, heart, intestines, eye, and brain tissues. Strong correlations were found between ET and its putative metabolites - hercynine, ET-sulfonate (ET-SO_3_H), and S-methyl ET. Hercynine accumulates in the brain after prolonged ET administration. This study demonstrates the uptake and distribution of ET and provides a foundation for future studies with ET to target oxidative damage in a range of tissues in human diseases.

## Introduction

**Figure 1:**
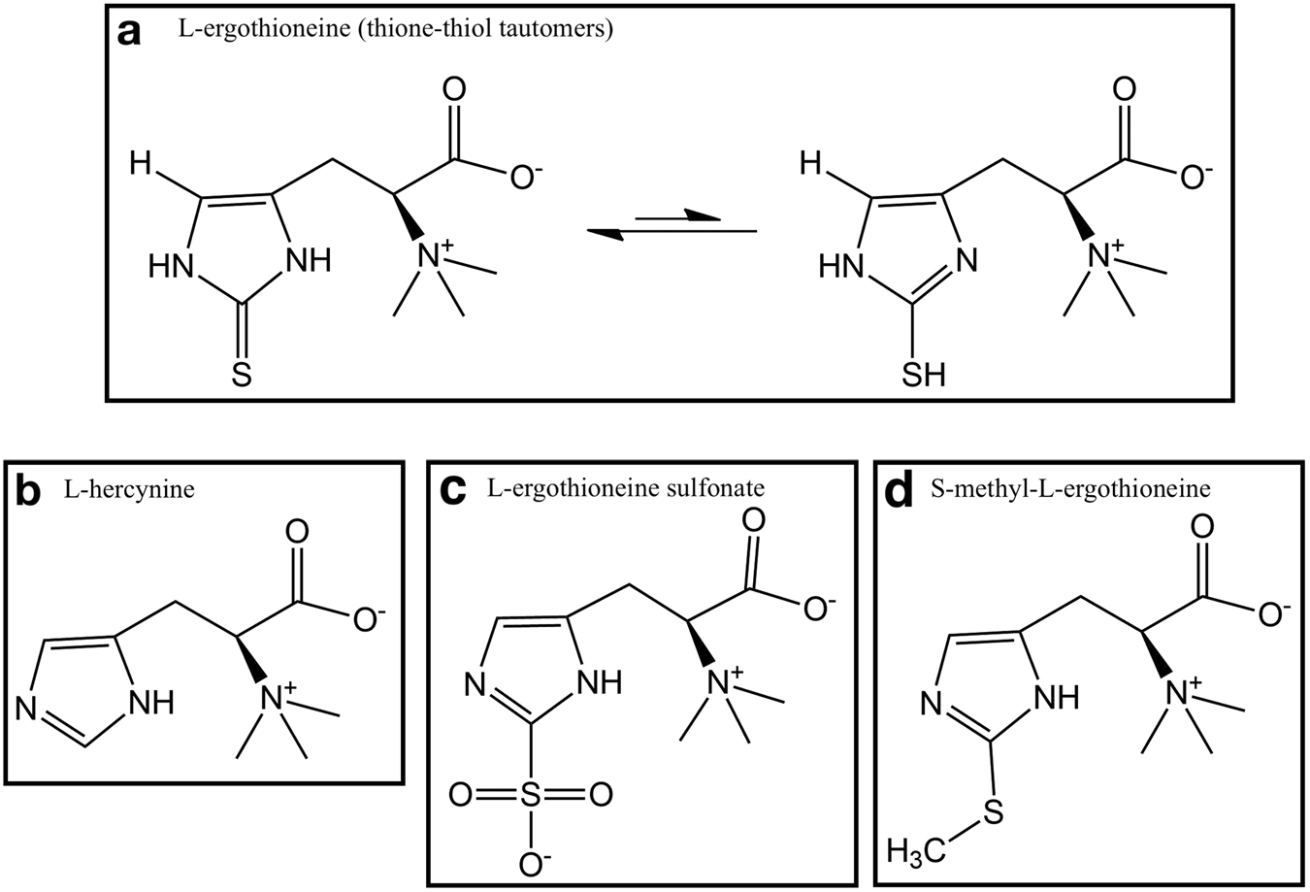
Structure of ergothioneine (ET) and its possible metabolites. (**a**) ET exists predominantly in the thione form at physiological pH. Putative metabolic derivatives of ET – (**b**) hercynine, (**c**) ET sulfonate (ET-SO_3_H), and (**d**) S-methyl ET.

L-ergothioneine (2-mercaptohistidine trimethylbetaine, C_9_H_15_N_3_O_2_S; ET) was discovered by Charles Tanret in the ergot fungus *Claviceps purpurea*^1^. ET exists as a tautomer between the thione and the thiol form (Fig. 1a), with the thione form dominating at physiological pH, providing greater stability *in vivo* than other low molecular weight thiols such as glutathione^2^. Biosynthesis of ET has been described in many fungi^3–5^ and various bacteria^6–11^. To date, biosynthesis of ET has not been detected in any animals or higher plants^12^. Despite the inability to biosynthesise ET, various animal and human tissues have been shown to accumulate ET at high concentrations^12–14^. This uptake of ET from dietary sources was found to be due to the transporter OCTN1 (organic cation transporter novel type-1) encoded by *SLC22A4*^14^. Silencing the *SLC22A4* gene was shown to prevent ET uptake in murine tissues^15^.

ET has been found at high levels in many foods, but levels are especially high in mushrooms where it can be synthesized^16–18^. In the body, the erythrocytes, bone marrow, liver, kidney, seminal fluid and the lens of the eyes are claimed to be rich in ET, possibly due to their predisposition to oxidative stress^14,19–22^. The high retention and long turn-over could be due to renal reabsorption and low urinary excretion^13^. Surprisingly, even with such avid retention and nearly ubiquitous distribution throughout the body, there are no reports of symptoms due to ET deficiency, although some studies have demonstrated a predisposition to stress^12^.

Although the physiological function of ET has yet to be fully established (reviewed in^12^ and^23^), many *in vitro* studies have demonstrated the ability of ET to scavenge hydroxyl radicals, hypochlorous acid, peroxynitrite^24^, and singlet oxygen^25^, modulate inflammatory responses^26–28^ and protect against UV and gamma radiation^29–31^. Conversely, OCTN1 knockout mice are more prone to oxidative stress^15^. Our previous work demonstrated that ET accumulates in the fibrotic liver of a guinea pig model of non-alcoholic fatty liver disease, which correlates with the progression of liver damage, suggesting a possible stress response by the injured tissue to suppress oxidative damage and delay further tissue injury^23,32^. Other studies have shown that ET can protect neurons both *in vitro* and *in vivo* against a range of stressors^33,34^, and declining blood levels of ET levels in subjects with mild cognitive impairment^35^ and Parkinson’s disease^36^, suggest that ET could be a factor for neuroprotection. Hence, there is growing interest in ET as a potential therapeutic compound for various conditions^37–39^.

Despite the established free radical scavenging properties of ET, little is known about its oxidation products. Servillo *et al*.^40^ investigated the possible oxidation products of ET and suggested that hercynine (Fig. 1b) and ET sulfonate (Fig. 1c; ET-SO_3_H) are the stable oxidation products of ET. Hercynine is also known to be an intermediary precursor of the ET biosynthetic pathway in the bacterial and fungal organisms mentioned earlier. S-methyl ET (Fig. 1d) could be generated through the methylation of the sulfur. Hercynine and S-methyl ET have been detected in human blood and urine, with high correlation to ET concentrations in the body^13,41^.

**Figure 2:**
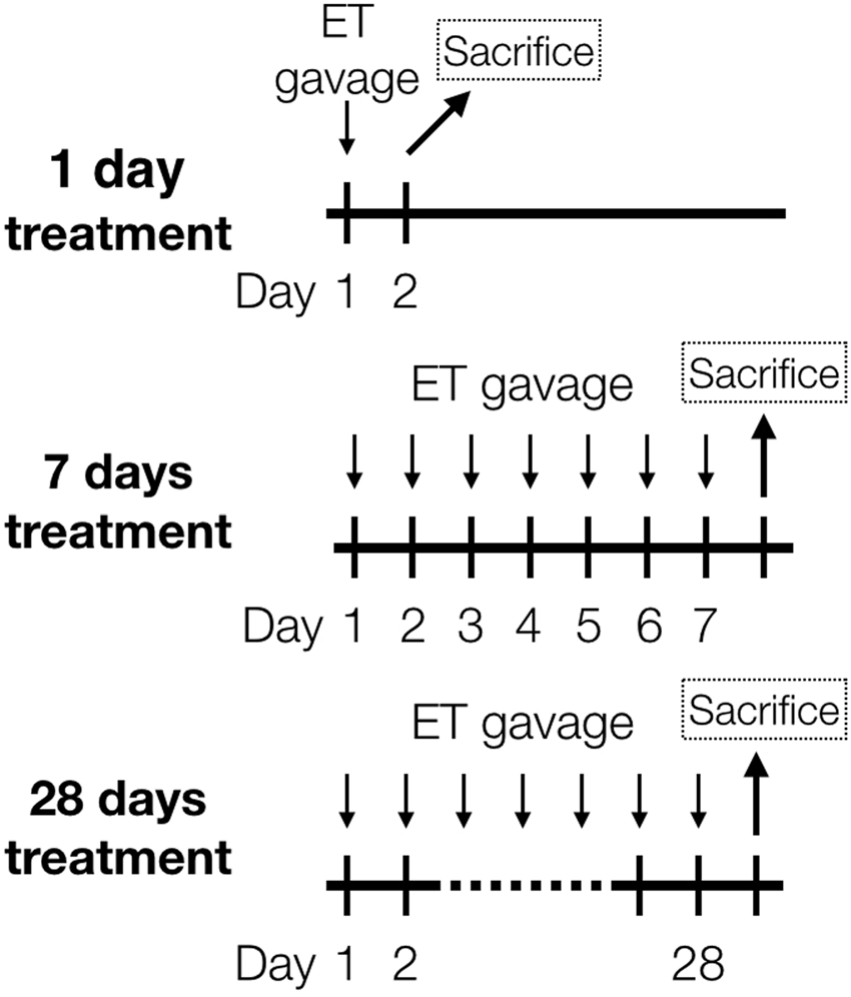
Experimental setup. C57BL6-J mice (n = 7) were orally gavaged with 35 mg/kg (ET+) or 70 mg/kg (ET++) of ET every day for 1, 7 and 28 days. Control mice were gavaged with saline. Mice were euthanized 24 h after the last administration, and organs were harvested.

Our previous clinical study investigated the uptake and pharmacokinetics of ET in healthy human subjects, demonstrating that ET is avidly absorbed and retained by the body following oral administration^13^, but of course the ability to sample human tissue is limited. The present study expands this work to detail the uptake and distribution of ET and related metabolites in various organs and tissues in the mouse by liquid chromatography tandem mass spectrometry (LC-MS/MS), following oral administration of pure ET over time (Fig. 2).

## Results

### Basal ET distribution in mice tissues

**Figure 3:**
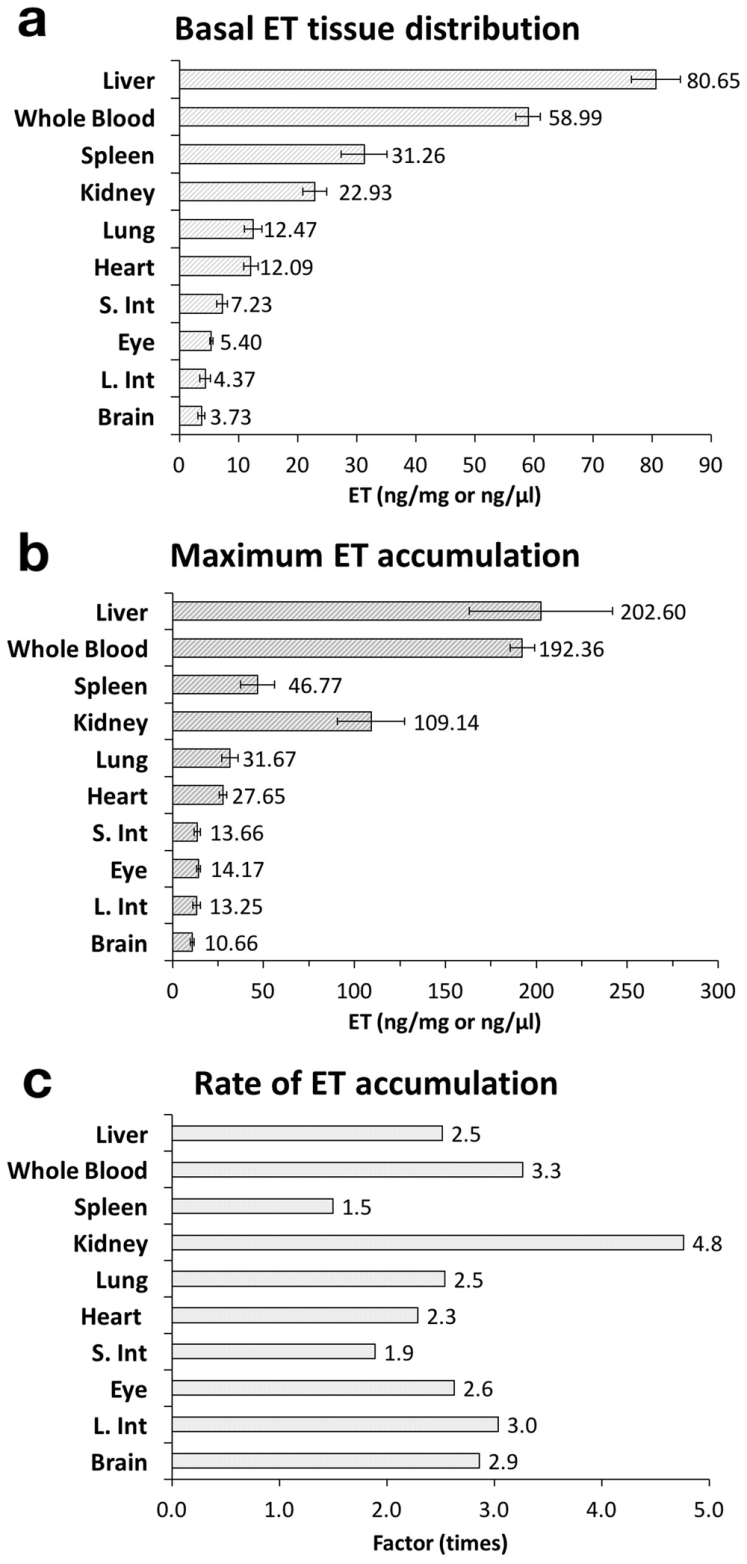
Distribution and accumulation of ET in mouse liver, whole blood, spleen, kidney, lung, heart, small intestine (jejunum/ileum), eye, large intestine, and brain (cortex). (**a**) Basal ET concentration in mice administered with single dose of saline (1-day control), n = 7. (**b**) Maximum ET accumulation in mice fed with 70 mg/kg/day ET for 28 days, n = 7. (**c**) Rate of ET accumulation was determined by calculating the fold change between maximum and basal ET concentrations. All values are expressed with standard error.

Despite not supplementing with ET, tissues of the control mice (administered saline) contained ET. Assessing the tissue ET concentrations of control mice, we found that of the 10 tissues measured (expressed in mass per wet weight of tissue ± standard error), liver had the highest amount of ET (80.65 ± 4.14 ng/mg), followed by whole blood (58.99 ± 2.05 ng/µl blood), spleen (36.45 ± 3.72 ng/mg), kidney (27.61 ± 2.61 ng/mg), lung (19.56 ± 1.46 ng/mg), heart (12.09 ± 1.20 ng/mg), small intestine (7.23 ± 0.86 ng/mg), eye (5.40 ± 0.29 ng/mg), large intestine (4.37 ± 0.93 ng/mg) and brain (3.73 ± 0.59 ng/mg) (Fig. 3a). As we detected ET in their standard diet (5.63 ± 0.33 ng/mg), we believe that the ET accumulates from the mouse diet, possibly over a long period of time.

### ET uptake after oral dosing

**Figure 4:**
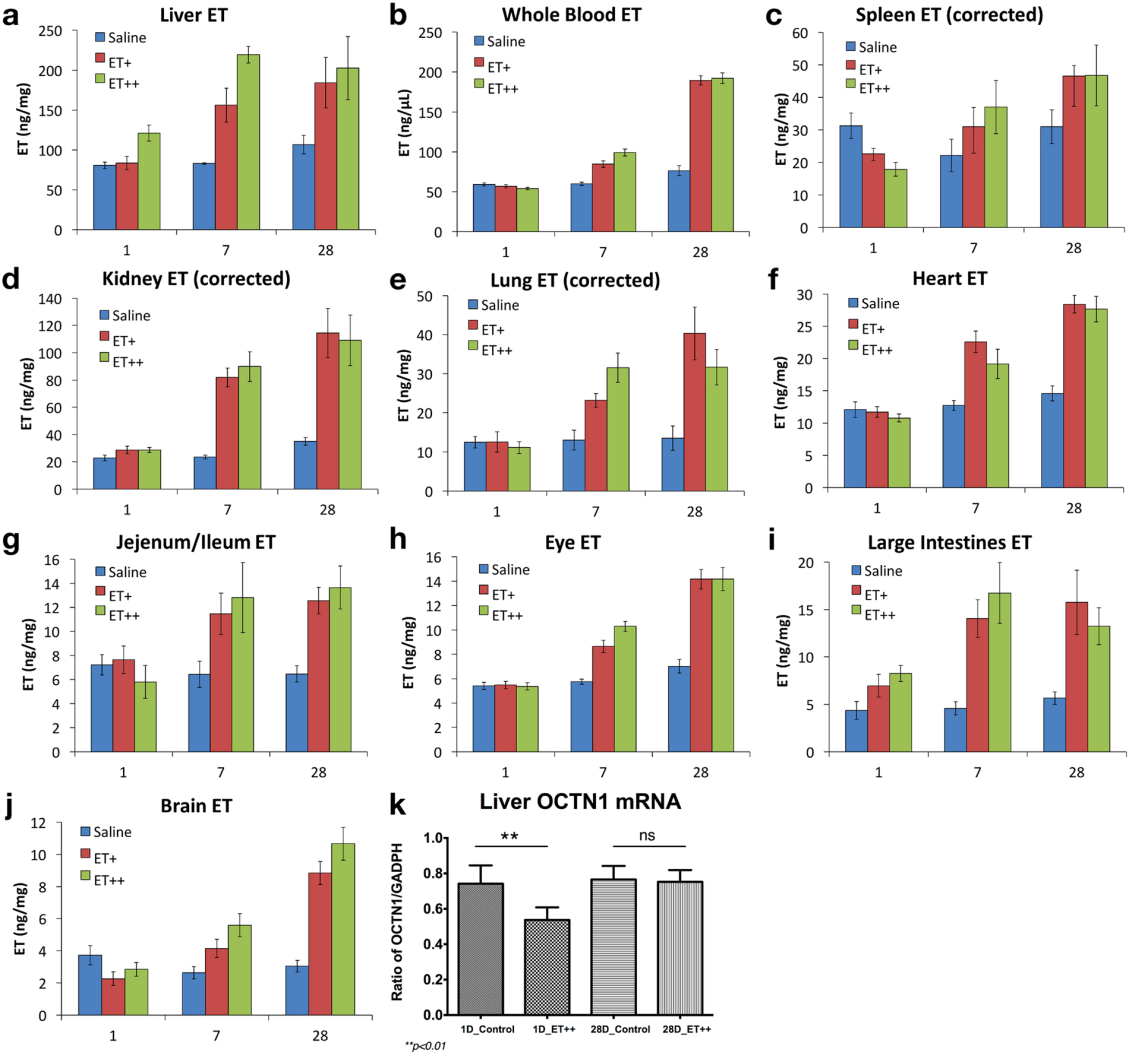
Uptake and accumulation of ET in various mouse tissues. (**a**–**j**) Mice (n = 7) were orally administered with either saline, 35 mg/kg (ET+) or 70 mg/kg (ET++) of ET per day for 1, 7 or 28 days, and 10 tissues (liver, whole blood, spleen, kidney, lung, heart, small intestine (jejunum/ileum), eye, large intestine, and brain) were harvested and ET concentration measured by LC-MS/MS. Spleen, kidney, and lung data were corrected for blood ET contribution. (**k**) RT-PCR of OCTN1 mRNA expression in liver of mice fed with saline (Control) or 70 mg/kg/day ET (ET++) for 1 and 28 days (n = 7); Two-tailed Mann-Whitney test **p < 0.01. All values are expressed with standard error.

Mice were administered with either saline, 35 mg/kg/day (ET+) or 70 mg/kg/day (ET++) of ET *via* oral gavage, for either 1 day, 7 days or 28 days. The levels of ET in the various tissues were measured by LC-MS/MS and presented in Fig. 4. After a single dose of ET (1 day, ET++), only the liver and large intestine tissues showed significant increases in tissue ET versus controls, while spleen ET levels decreased. (Fig. 4 and Supplementary Table 1). After 7 days of feeding, significant increases in ET levels were observed in all the tissues examined (Supplementary Table 1). Modest increases (about 2-fold with respect to controls) in ET were observed in whole blood, brain, eye, heart and spleen, meanwhile liver, kidney, lung and intestinal tissue levels were considerably higher (Fig. 4). To ensure accurate measurement, intestinal content had been completely washed away before analysis of the small and large intestinal tissues.

Statistical analysis revealed that 35 mg/kg/day ET for 7 days is sufficient to bring about a modest but significant elevation in ET levels in most tissues (Supplementary Table 2), while 70 mg/kg/day ET for 7 days effectively doubles the amount of ET accumulated in all tissues except the spleen (Fig. 4). We noted that the control animals after 28 days of feeding had small but significantly higher ET levels in whole blood and kidney than the 1 and 7 days animals (Fig. 4, Supplementary Table 2). In addition, there was no statistical difference between the low and high ET dose in all tissues except the liver and eye (Supplementary Table 3).

### Maximal accumulation of ET in tissues

Interestingly, peak ET accumulation was reached in some tissues as ET levels plateaued by 28 days of feeding at both ET concentrations (Fig. 4). ET levels in small intestine (jejunum/ileum) and large intestine seems to have reached their maximal levels after 7 days of ET+ dosing, while liver and lung required 7 days of ET++ dosing to reach their maximum concentration, with all having no significant differences compared to ET levels at 28 days of feeding (Fig. 4, Supplementary Table 2). We also evaluated the mRNA expression of the ET transporter, OCTN1, in the liver after 1 and 28 days administration, and found that OCTN1 expression was reduced after a single dose of ET++ (Fig. 4k). However, there was no significant difference in OCTN1 mRNA expression between control and ET++ groups at 28 days (Fig. 4k).

We analysed the maximal ET accumulation in other tissues at high dose (ET++) for 28 days and found that, like ET tissue distribution in control animals, the liver and whole blood remain as the highest reservoirs for ET accumulation (Fig. 3b). Compared to basal condition, the kidney has greater accumulation of ET than spleen (Fig. 3b). Of all tissues, the kidney has the greatest overall increase in ET concentration with a 4.8-fold increase in kidney ET at 28 days of high dose (ET++) relative to control, while liver and whole blood of the same group only increased by 2.5- and 3.3-fold relative to control animals (Fig. 3c).

### Contribution of blood in measuring tissue ET and hercynine levels

Since erythrocytes are known to accumulate ET, whole blood hemoglobin levels were measured as an indicator of total erythrocytes and found to be fairly constant between the mice (Supplementary Data 1a), with no significant correlations with whole blood ET (Supplementary Data 1b), suggesting that any ET accumulation in whole blood is not due to an increase in the number of erythrocytes.

As whole blood possesses high levels of ET, we investigated whether the ET detected in the tissues was present within the tissue itself or resulted from blood remaining in the tissue after washing. Several samples of liver and brain tissues were used to estimate the amount of blood contamination via fluorescent quantification of hemoglobin (Supplementary Table 4). We calculated that only minute amounts of blood were present in the liver and brain tissue, and after adjusting for this, the resulting ET levels were only slightly reduced by 1% and 5% respectively (Supplementary Table 4). As the lungs, spleen and kidneys are known to be highly vascularized, we measured the blood content in these tissues, and found their hemoglobin levels to be fairly consistent between the samples (Supplementary Data 2). As a result, we observed that after subtracting the ET or hercynine from contaminating blood, there is no significant difference in the pattern of ET or hercynine accumulation (Supplementary Data 2). We calculated that blood ET contributed approximately 33%, 24%, and 13% for total lung, spleen and kidney ET respectively, while blood hercynine contributed approximately 19% for lung and spleen, and 13% for kidney (Supplementary Data 2p). Hence, all lung, spleen, and kidney ET and hercynine data reported in this paper are corrected for blood contamination.

### Tissue distribution of hercynine, ergothioneine sulfonate and S-methyl ergothioneine

**Figure 5:**
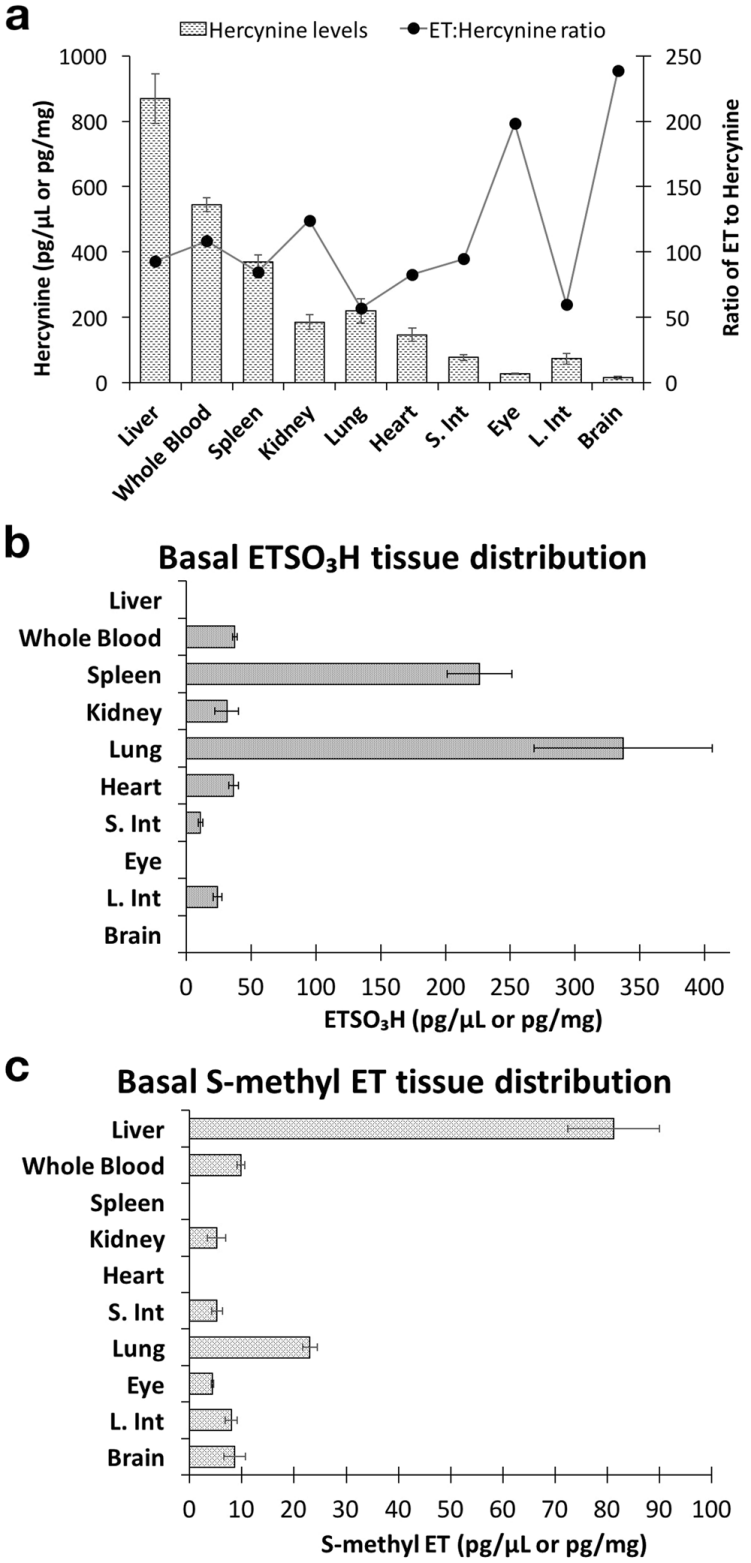
Basal tissue distribution of ET related metabolites in mice fed with single dose of saline (1-day control), n = 7. (**a**) Hercynine and ratio of ET to hercynine concentration, (**b**) ET-SO_3_H concentrations, and (**c**) S-methyl ET concentrations. All values are expressed with standard error.

Hercynine was detectable in all the tissues studied (Fig. 5a). We found that basal hercynine levels were approximately 100 times lower than ET concentrations in the respective tissues. Like ET, the highest levels of hercynine were found in liver (869.10 ± 76.53 pg/mg), followed by whole blood (544.53 ± 21.02 pg/µl), spleen (368.92 ± 22.13 pg/mg), kidney (184.81 ± 22.45 pg/mg), lung (219.11 ± 36.63 pg/mg), heart (146.24 ± 21.27 pg/mg), small intestine (jejunum/ileum; 76.33 ± 8.09 pg/mg), large intestine (72.90 ± 16.88 pg/mg), eye (27.26 ± 2.21 pg/mg) and brain (15.62 ± 3.84 pg/mg) (Fig. 5a). We tabulated the ratio of ET to hercynine concentrations, and discovered that while most of the tissues have a ratio of 100:1, brain and eye have much lower levels of hercynine with a ratio of about 200:1, while large intestine and lung had a ratio of about 60:1 (Fig. 5a).

Despite being a potential oxidation product of ET, ergothioneine sulfonate (ET-SO_3_H) was only detectable in 7 of the 10 tissues measured (Fig. 5b). Interestingly, even though the liver and whole blood have the highest amount of ET, ET-SO_3_H was not detectable in the liver and present at very low levels in whole blood. In addition, no ET-SO_3_H was detectable in the eye and brain. Highest levels of ET-SO_3_H were found in the lung (337.45 ± 68.71 pg/mg) and spleen (226.23 ± 25.21 pg/mg), while the concentrations in the remaining tissues fall within the range of 10 to 37 pg/mg or pg/µl (Fig. 5b).

On the other hand, S-methyl ET was detectable in all tissues except the spleen and heart (Fig. 5c). Highest levels of S-methyl ET were found in the liver (81.23 ± 8.80 pg/mg), followed by lung (23.01 ± 1.41 pg/mg), with the concentrations in other tissues ranging from 4 to 10 pg/mg or pg/µl (Fig. 5c).

### Levels of hercynine, ergothioneine sulfonate and S-methyl ET following ET administration

**Figure 6:**
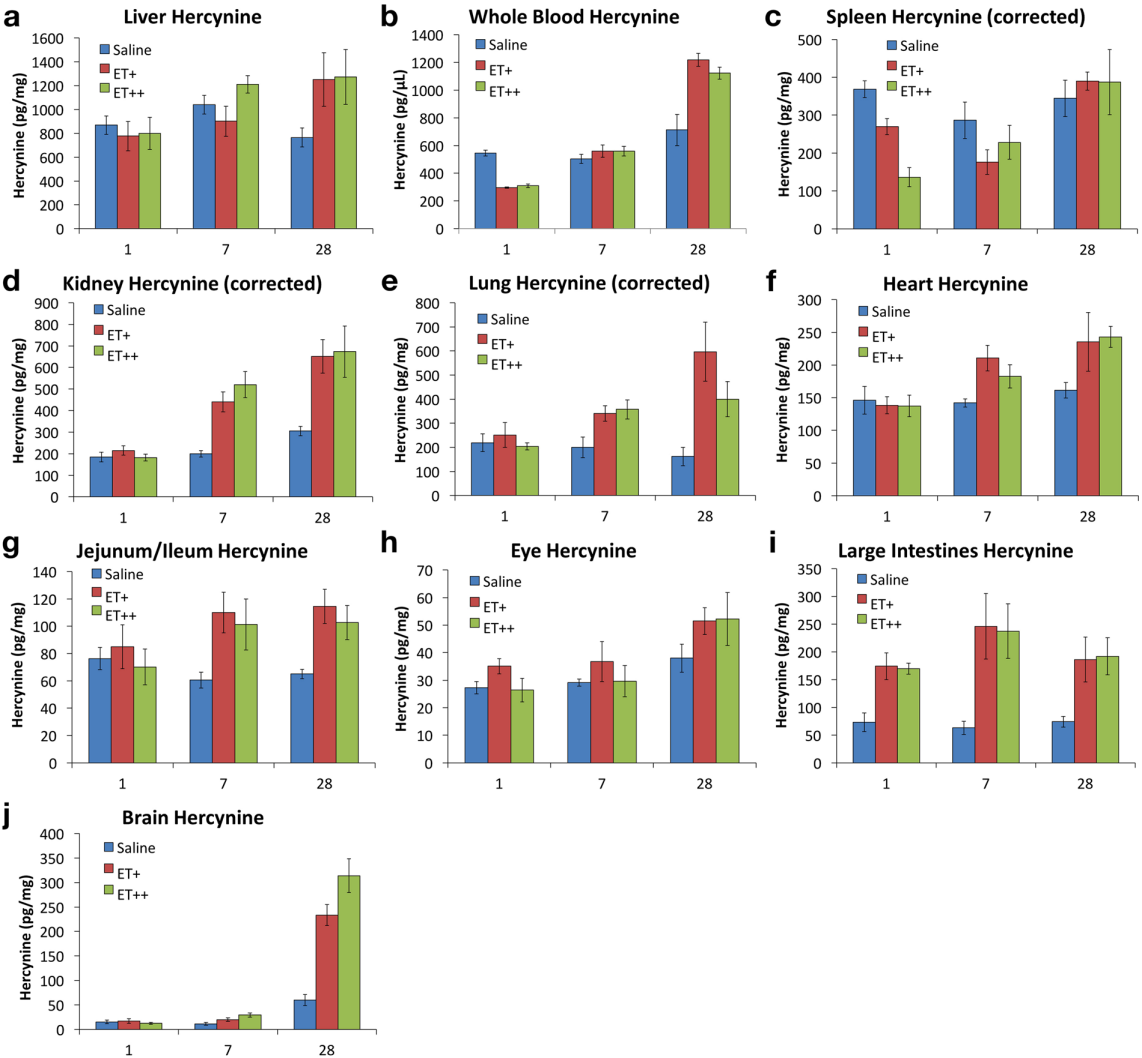
Accumulation of hercynine in mouse tissues. (**a**–**j**) As per ET concentration quantified in Fig. 4, hercynine levels were determined simultaneously by LC-MS/MS in liver, whole blood, spleen, kidney, lung, heart, small intestine (jejunum/ileum), eye, large intestine, and brain. Spleen, kidney, and lung hercynine data were corrected for blood hercynine contribution. All values are expressed with standard error.

Interestingly, after a single dose of ET, we noted a decrease in hercynine levels in whole blood and spleen, but an increase in the large intestine (Fig. 6). However, after 7 days of ET administration, whole blood hercynine levels were similar to the control mice, while spleen hercynine levels still remained lower than controls. With continued administration, hercynine levels began to increase in most tissues, and like ET, appeared to peak by 28 days. Hercynine concentrations in the small intestines (jejunum/ileum) plateaued after 7 days, while that of large intestine were noticeably high from the first day (Fig. 6). Notably, despite having the lowest basal levels of hercynine, brain hercynine levels significantly increased with ET administration, reaching 5 to 6-fold of the controls after 28 days, which was not observed in any other tissue examined (Fig. 6j).

**Figure 7:**
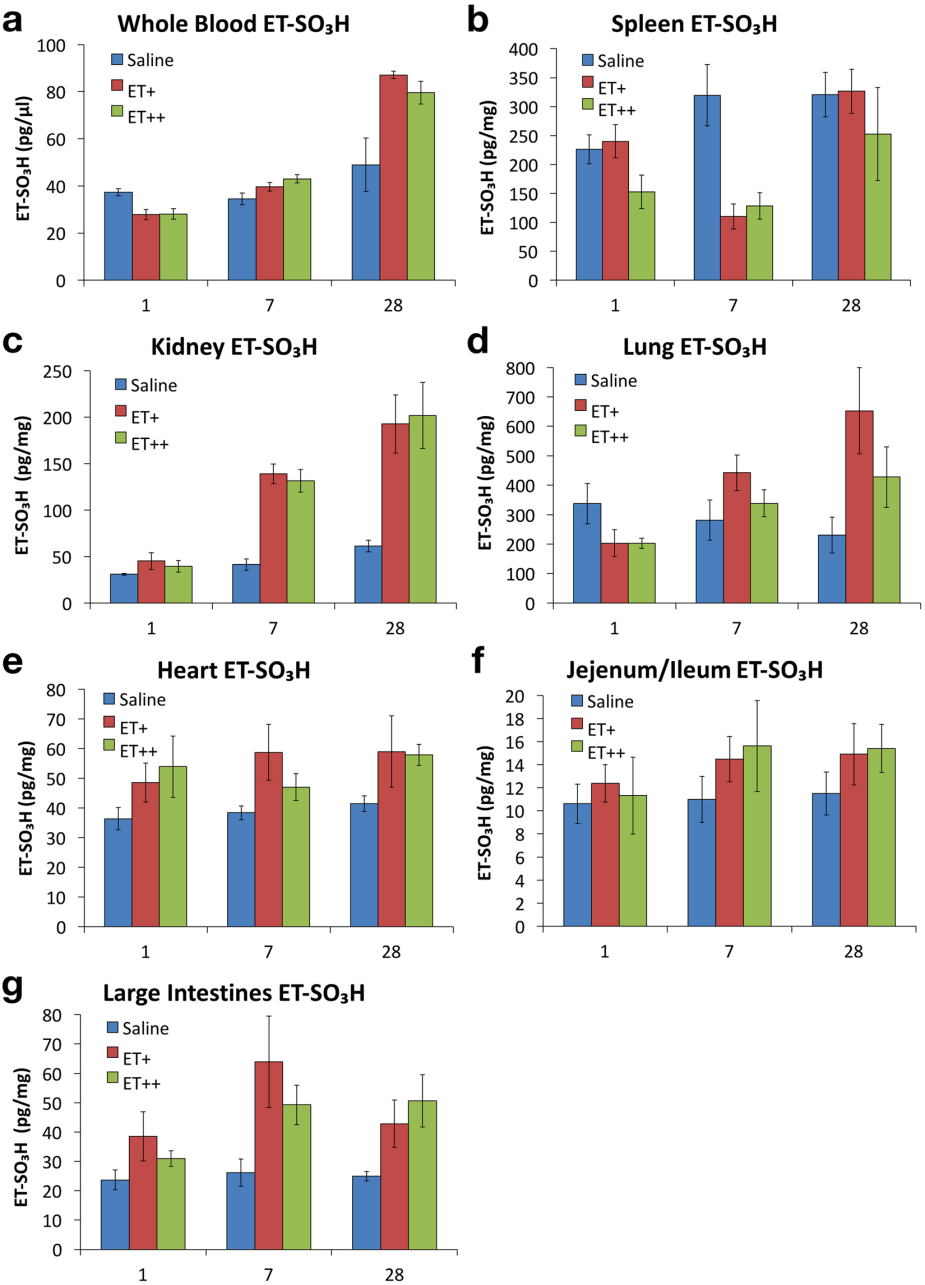
Accumulation of ergothioneine sulfonate (ET-SO_3_H) in mouse tissues. (**a**–**g**) Similar to hercynine, ET-SO_3_H levels were quantified by LC-MS/MS in whole blood, spleen, kidney, lung, heart, small intestine (jejunum/ileum), and large intestine. All values are expressed with standard error.

ET-SO_3_H concentrations were found to be decreased in whole blood, spleen and lung after the first dose of ET (Fig. 7). Spleen ET-SO_3_H levels were further decreased at 7 days, but returned to levels found in control mice after 28 days. Whole blood, heart and intestinal ET-SO_3_H levels appeared to increase after 7 and 28 days ET administration. Despite having high levels of ET-SO_3_H, lung ET-SO_3_H levels did not show any clear trends with respect to the frequency and dosage of ET. Following kidney ET levels, kidney ET-SO_3_H levels significantly increased after 7 and 28 days ET administration (Fig. 7).

**Figure 8:**
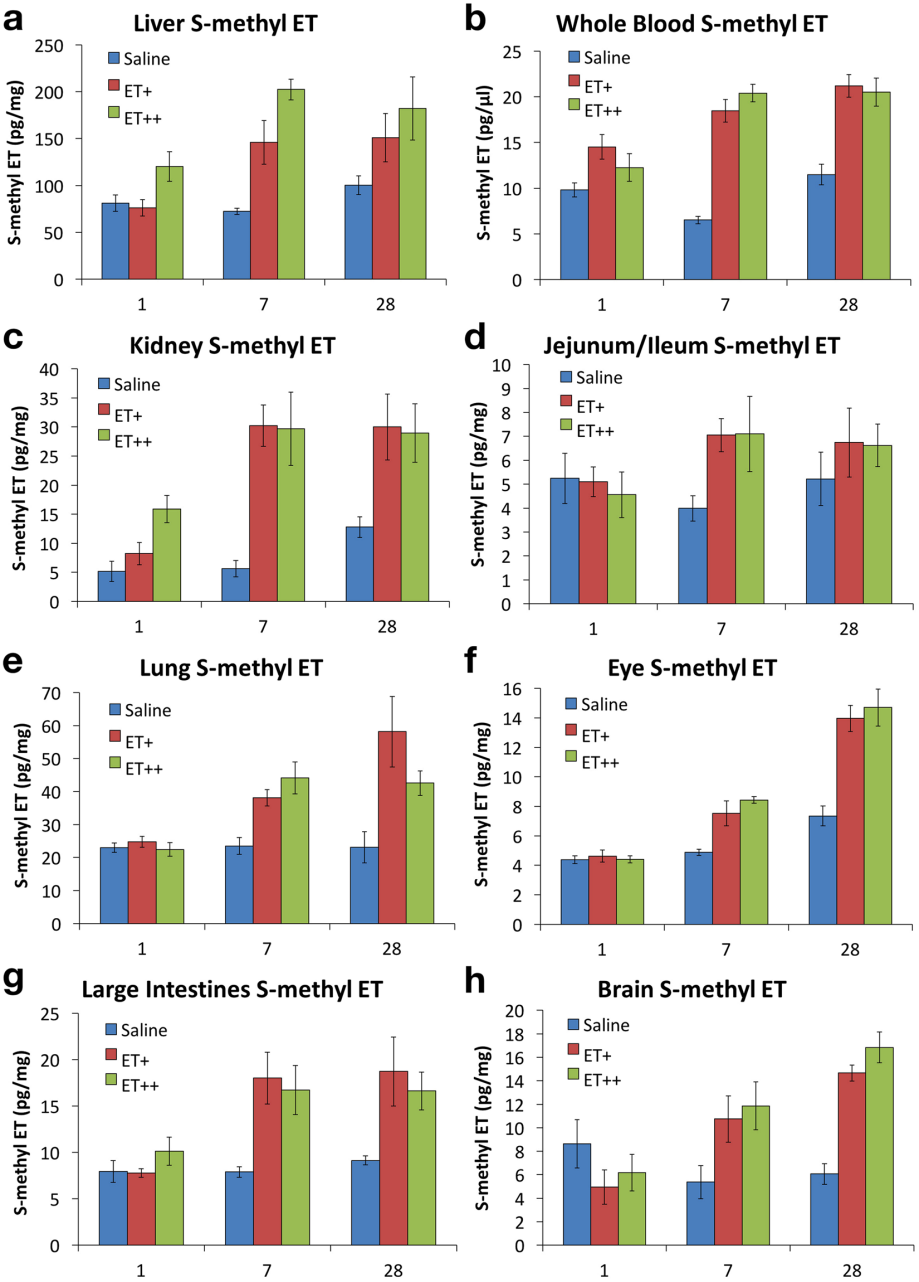
Accumulation of S-methyl ET in mouse tissues. (**a**–**h**) Similar to hercynine, S-methyl ET levels were quantified by LC-MS/MS in whole blood, spleen, kidney, lung, heart, small intestine (jejunum/ileum), and large intestine. All values are expressed with standard error.

S-methyl ET followed a similar tissue accumulation trend with that of ET (Fig. 8), with tissue levels of S-methyl ET increasing after 7 and 28 days of ET administration. S-methyl ET levels in the liver and kidney increased after a single dose of ET, and continued to increase significantly in kidney after 7 and 28 days of treatment. Except for the liver, no significant differences in tissue S-methyl ET were seen between the low and high doses of ET.

### Correlation of ET with hercynine, ergothioneine sulfonate and S-methyl ET

**Figure 9:**
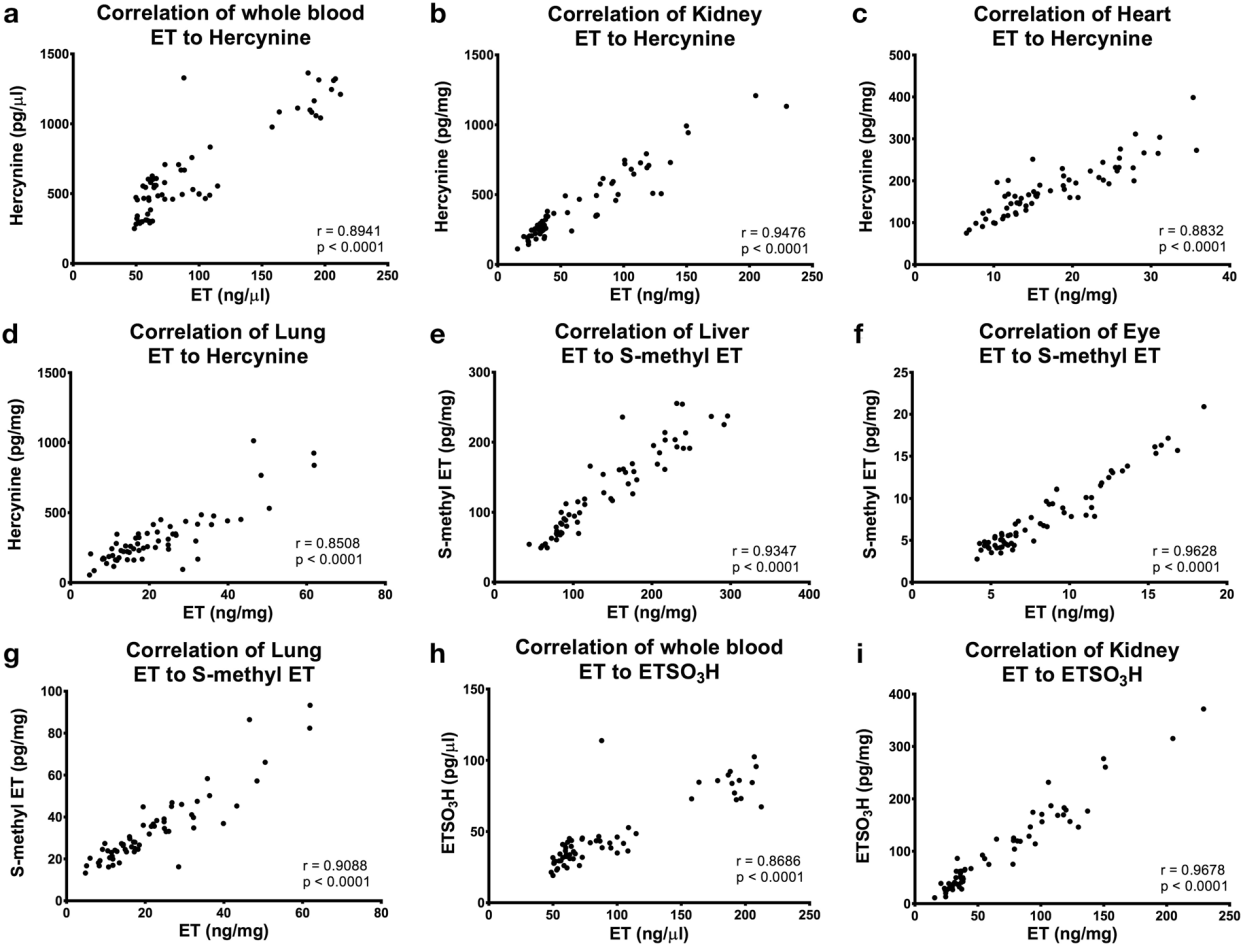
Correlation of ET concentration with hercynine, ET-SO_3_H, and S-methyl ET. Other correlations can be found in Supplementary data 3. (**a**–**d**) Correlation of whole blood, kidney, heart and lung ET to hercynine. (**e**–**g**) Correlation of liver, eye and lung ET to S-methyl ET. (**h**–**i**) Correlation of whole blood and kidney ET to ET-SO_3_H. All values are expressed with standard error.

We noticed that the concentration of the suggested ET metabolites followed similar trends to that of ET in the various tissues, and therefore examined any correlations between them (Fig. 9, Supplementary Data 3). Using all collated data, we found that ET levels correlates well with hercynine in whole blood, kidney, heart and lung (Fig. 9a–d), while S-methyl ET correlate well with liver, eye and lung ET (Fig. 9e–g). On the other hand, ET-SO_3_H levels were only found to correlate with whole blood and kidney ET (Fig. 9h and i). We also tabulated the correlation between whole blood ET and the rest of the organs and found that other than lung and the intestines, blood ET correlates well with ET levels in other tissues (Supplementary Data 3).

## Discussion

Some studies into the tissue distribution of ET were undertaken in the 1950–1980s (extensively reviewed in^12^). However, these mainly utilised chemical assays, or at best UV-liquid chromatography quantification, to estimate tissue ET levels, methods which lack sensitivity and specificity^12^. To our knowledge, our study is the first to examine the uptake and distribution of ET in mouse tissues before and after oral administration of pure ET (Tetrahedron, France), and utilising LC-MS/MS to accurately quantify the accumulation of ET over time. The higher sensitivity of the instrument allows the detection of ET in tissues such as murine brain and human plasma^13^, which could not be detected in the past^19^.

Given that the average mass of a mouse is 20 g, the mice were fed about 0.7 mg and 1.4 mg of ET per day, corresponding to 35 mg/kg/day and 70 mg/kg/day, respectively. Many groups found that ET levels in mushrooms vary considerably, with a median value of about 1 mg/g^18^. To put this into perspective, this equates to around 0.7–1.4 g of mushrooms, or about 3.5% to 7% total body mass of an average mouse per administration. Nonetheless, even at such high doses no negative side effects of ET were noted in the animals. The levels of ET in the chow are low, and given that C57B6J mice consumed about 4 grams of food per day^42^, the amount of ET from the mice chow is very low compared to the amount we administered. Nevertheless, significant basal levels were present, consistent with efficient uptake and accumulation of ET.

Basal ET levels were found to be highest in the liver and whole blood, being approximately 10 to 20-fold higher levels than the brain and eye, suggesting that these tissues may be a primary reservoir for ET in the body. Due to the small volume of blood collected, we were unable to separate the blood plasma from the erythrocytes. Nevertheless, our human studies^13^ and others^43,44^ demonstrated that in blood, ET is predominantly present in the erythrocytes rather than in the plasma. As discussed by Cheah & Halliwell^12^, ET is suggested to be preferentially distributed in tissues that are exposed to high degrees of oxidative stress. The high levels of ET accumulated in liver and erythrocytes may have a pivotal role in protecting cells from oxidative damage. For instance, earlier studies found that ET reduces ferrylmyoglobin into metmyoglobin, thus inhibiting lipid peroxidation and protected against tissue injury during ischemia/reperfusion^45,46^. Furthermore, ET can lower nitrite-induced oxidation of haemoglobin in isolated rabbit blood^47^. Another study suggested that ET may also play a role in erythropoiesis^48^. Nonetheless, despite the lack of an established function of ET, the avid accumulation and retention suggests the importance of this compound, which may help maintain the metabolic activity and protect against potential oxidative damage in tissues.

The ET uptake by different tissues varied greatly in both overall accumulation (i.e. 1.7-fold increase in spleen, relative to 4.8-fold in the kidney after 28 days), and rate of uptake (accumulation in liver and lung reached peak levels prior to the brain and heart). Only the liver and large intestine showed significant ET elevations after a single administration, which suggests that the liver preferentially stores ET following intestinal absorption, even prior to erythrocyte accumulation. Interestingly, a recent study in a mouse model of chronic kidney disease revealed that these animals had lower intestinal absorption of ET, which led to a significant decline in blood ET but not liver ET levels^49^. This suggests that the body preserves liver ET levels.

The rapid accumulation in the large intestine highlights the possible importance of ET in the body to warrant such a response. After administration for 7 days, all tissues demonstrated a two-fold or greater increase in the amount of ET. These data will be valuable for determining dosage of ET to be used in future studies.

Despite doubling the amount of ET administered between low and high dose (35 to 70 mg/kg/day), no significant differences in ET accumulation were observed, except in the liver and eye. This could be due to variation in ET absorption between the animals resulting from variations in OCTN1 expression. Alternatively, the ET accumulation might not be homogenous throughout the organs, hence differing sections of the tissue cut for ET quantification may affect the results to some extent. Dose-dependent accumulation of ET in the eye could suggest an important role in providing protection, since oxidative stress in the eye is common in ocular diseases^50,51^. Antioxidants such as resveratrol have been tested on various eye diseases^52^, but no studies have been done with ET. ET can be found at different concentrations in various parts of bovine and porcine ocular tissues^20^, and one study showed that ET can protect corneal endothelial cells against oxidative stress and unfolded protein response^53^.

Due to difficulties in obtaining a urine sample at specific time points, the excretion of ET was not determined in this project, and hence little is known about the retention of ET by the mice. However, high accumulation of ET in the large intestine, as well as the significant rate of accumulation of ET in the kidney may imply that ET is avidly absorbed and retained by the body. Similarly, other studies have shown that ET is avidly retained by cells and tissues with minimal excretion due to renal reabsorption^15^. Our ET human studies revealed minimal excretion of ET in urine, indicating that ET is predominantly retained by the body after oral administration^13^. In rats, the *in vivo* half-life of ET was determined to be around one month^48^.

Despite the rapid ET accumulation in liver, lung, small intestine (jejunum/ileum) and the colon, it appears that these tissues became saturated after repetitive doses for one week, with no further increase in ET with administration up to 28 days. Without an earlier timepoint, it may be possible that the uptake has reached peak levels before the 7^th^ day. Although the initial (after a single dose) decrease in mRNA expression of the ET transporter OCTN1 in the liver, the expression levels returned to normal (same as controls) after 28 days. Hence, even with normal OCTN1 expression levels, the lack of further increase in ET uptake after 28 days may suggest an equilibrium has been reached between the uptake and excretion and/or utilisation of ET by the liver.

ET was also found to accumulate in the brain (cortex), albeit at much lower levels compared to the other organs (likely due to the slower passage through the blood brain barrier). Nevertheless, the 2.9-fold increase in ET levels after 28 days of administration relative to controls, suggests a slow but gradual accumulation of ET, with no certainty that a peak was reached in this time. The function of ET in the brain is uncertain, however OCTN1 expression had been observed in the brain and various neuronal cells^54,55^. The ability of ET to permeate the blood brain barrier could indicate a functional role of ET in the central nervous system and neurons. ET had been demonstrated to protect rat pheochromocytoma cells against β-amyloid-induced cell death^56^, as well as protect neurons from N-methyl-D-aspartate excitotoxicity^33^. Also, ET could be important for mouse neuronal differentiation^57^. Our recent study demonstrated that blood levels in mild cognitively impaired subjects were significantly lower than age-matched healthy individuals^35^, and likewise a study in Parkinson’s disease patients revealed lower levels of serum ET^36^. Taken together, these could suggest that ET may play a protective role in the brain.

The drop in ET levels in the spleen upon a single administration remains a mystery. As a major organ for lymphocytes development and erythrocytes homeostasis, the spleen has been known to accumulate ET^58,59^. Since ET is found in high concentrations in erythrocytes, the spleen could be a site for the recycling of ET from worn out cells.

While OCTN1 transports primarily ET^14^, it has been shown to transport hercynine at a 25-fold lower rate than ET^60^. It is currently unknown if it can transport the other metabolites of ET. Of the three ET-related metabolites assessed, only hercynine was detectable and quantifiable in all the tissues analysed, and displayed significant correlation with ET concentrations in the respective tissues. This likely indicates that hercynine is indeed a metabolic product of ET in the body, as was similarly observed in our human study^13^. Hercynine is known to be a precursor in ET biosynthesis, and some groups have postulated that hercynine is an intermediate product in the oxidation pathway of ET^40,61^. In particular, Sevillo *et al*. hypothesised that the oxidation of ET involves either a disulfide intermediate (similar to glutathione and glutathione disulfide), which is hydrolysed back to ET, generating ET-SO_3_H and hercynine as end products, or ET is directly oxidized into ET-SO_3_H as a stable end product^40^. However, in our present study, the accumulation of ET does not always correlate with the levels of these suggested oxidative products. For example, the liver had the highest amount of ET and yet had no quantifiable levels of ET-SO_3_H, while lung and spleen which had moderate levels of ET had the highest levels of ET-SO_3_H. Likewise, in the brain, despite having the lowest levels of tissue ET, there was an appreciable elevation in hercynine levels (far more than any other tissue). This poses a question as to how ET-SO_3_H or hercynine are generated in the lung, spleen and brain, and whether the conditions favor the accumulation of particular oxidation products. These tissues can be exposed to high levels of oxidative stress, which may explain the appreciable increase in the putative oxidation products.

Little is known regarding the formation of S-methyl ET. This metabolite could be easily detected in human blood and urine^13^, and most of the mouse tissues measured (except spleen and heart). S-methyl glutathione can form by reaction between glutathione and methyl halides involving glutathione-S-transferase^62^. To date however, no mechanism or enzymes are known to facilitate the methylation of ET.

## Conclusion

Our study presents the first data investigating the uptake of pure ET and distribution in various tissues and organs of the mouse using modern methodology. As with our previous human study^13^, these data showed that ET is well retained in the blood, and also indicates possible reabsorption by the kidneys, demonstrating the accumulation and retention by the body of this compound. While ET was highly accumulated in all tissues, a maximal accumulation in ET levels was observed in many tissues. The ability of ET to cross the blood brain barrier and accumulate in the brain tissues following oral consumption, highlights a possible function in the brain and also makes this compound a viable candidate for studies into neurodegenerative disorders. Overall our study provides a strong basis for future research into this unique compound, especially with respect to therapeutic efficacy in human disease and animal models of them.

## Material and Methods

### Chemicals and reagents

L-ergothioneine (ET; purity > 98%), ET sulfonate (ET-SO_3_H), L-hercynine, S-methyl ET, and heavy-labelled L-ergothioneine-d9 (ET-d9) and L-hercynine-d9 were provided by Tetrahedron (Paris, France; www.tetrahedron.fr). Stock solutions (1 mM) were prepared in ultrapure water (Arium pro, Sartorius AG, Gottingen, Germany) and stored at −20 °C. HPLC-grade acetonitrile and methanol, and MS graded formic acid were purchased from Fisher (Hampton, USA), GC-grade acetone from Tedia (Fairfield, USA), Triton X-100 from USB corporation (Cleveland, USA), and phosphate buffered saline (PBS)from Lonza (Basel, Switzerland). All other chemicals were obtained from Sigma - Aldrich (St Louis, USA) unless otherwise stated.

### Animal studies

Male C57BL6-J mice (Comparative Medicine Centre for Animal Resource, National University of Singapore) were housed randomly four to five per cage and allowed water and standard mice chow *ad libitum* in a barrier animal facility with a 12-hour light-dark cycle. Animals were cared for in accordance with criteria from the National University of Singapore, Institutional Animal Care and Use Committee (NUS IACUC). All the procedures were approved by the NUS IACUC, protocol number 082/12. All experiments were performed accordance with NUS guidelines and regulations.

### Administration of ET and collection of tissues

Mice (n = 7) were administered with either saline (0.9% *w/v* sodium chloride; control), 35 mg (ET+) or 70 mg (ET++) of ET (dissolved in saline) per kilogram bodyweight per day for a period of either 1 day, 7 days or 28 days, via oral gavage (Fig. 2). Mice were euthanised by CO_2_ asphyxiation 24 h after the final ET administration, followed immediately by cardiac puncture to collect whole blood in EDTA tubes. Whole blood aliquots (50 µl) were stored at −80 °C. Various organs were excised and washed with PBS, and then deionized water before snapped frozen in liquid nitrogen and stored at −80 °C. A portion of the liver tissue was kept in RNAlater solution (Life Technologies, CA, USA) at −20 °C to preserve RNA for extraction at a later point.

### Whole blood sample preparation for ET measurement

Aliquots of whole blood were thawed on ice and vortexed briefly to ensure homogeneity. 10 µl of whole blood were mixed with ET-d9 and hercynine-d9 internal standards and ultrapure water to 100 µl final volume. Samples were heated at 80 °C for 15 min to release protein-bound ET, then clarified by protein precipitation with 500 µl of cold acetone overnight at −20 °C. The precipitate was removed by centrifugation (20,000 *g*) and the supernatant was evaporated under a stream of N_2_, and reconstituted in ultrapure water. Samples were centrifuged to remove any debris before transferring into silanized glass inserts with vials (Agilent CrossLab) for LC-MS/MS analysis.

### Tissue sample preparation for ET measurement

Liver, brain (cortex), kidney, spleen and lung tissues were thoroughly washed in PBS and rinse in deionized water before snapped frozen. About 5–10 mg were homogenised with a motorized pellet pestle (Sigma-Aldrich) in 150 µl of deionized water containing internal standards. After homogenization, 750 µl of ice-cold methanol were added and vortexed for at least 30 s before incubating the samples overnight at −20 °C. The samples were centrifuged and supernatants were evaporated under a stream of N_2_, before reconstituting in ultrapure water. Any debris was removed by centrifugation before transferring to glass inserts for LC-MS/MS analysis.

For eye extraction, the whole eye was weighed and homogenized in ultrapure water. For the heart tissues, whole heart was first thawed and cut along the septum on a chilled plate, and washed thoroughly in PBS to remove excess blood. The tissue was then rinsed in deionized water before snap-freezing in liquid N_2_ and ground into fine powder using a mortar and pestle. About 5 mg of powdered tissue was accurately weighed before homogenization. For the small intestine (jejunum/ileum) and colon, a 1 cm portion was cut longitudinally and washed in PBS to remove all intestinal content before weighing and homogenization for analysis. All metabolites levels were normalised against the mass of tissue used, and expressed in mass per wet weight of tissue ± standard error.

### Liquid chromatography tandem mass spectrometry (LC-MS/MS) analysis

LC-MS/MS was carried out using an Agilent 1200 LC System coupled to an Agilent 6460 ESI tandem mass spectrometer. Samples were kept at 10 °C in the autosampler. 2 µl of the processed samples were injected into a Cogent Diamond-Hydride column (4 µm, 150 × 2.1 mm, 100 Å; MicroSolv Technology Corporation) maintained at 30 °C. Solvent A was acetonitrile in 0.1% formic acid, and Solvent B was 0.1% formic acid in ultrapure water. Chromatography was carried out at a flow rate of 0.5 ml/min using the following gradient; 1 min of 20% solvent B, following by a 3 min gradient increase of solvent B to 40% to elute ET-SO_3_H and ET. To elute hercynine and S-methyl ET, solvent B was increased to 90% in 1 min, and maintained for 3.5 min before returning to 20% for 3.5 min to re-equilibrate the column. The total run time was 12 min. The retention times for ET-SO_3_H, ET, hercynine and S-methyl ET were 3.6, 4.2, 6.8 and 6.9 min, respectively.

Mass spectrometry was carried out under positive ion, electrospray ionization mode, using multiple reaction monitoring (MRM) for quantification of specific target ions. Capillary voltage was set at 3200 V, and gas temperature was kept at 350 °C. Nitrogen sheath gas pressure for nebulizing sample was at 50 psi, and gas flow set at 12 L/min. Ultra-high purity nitrogen was used as collision gas. Precursor to product ion transitions and fragmentor voltages (V)/collision energies (eV) for each compound were as follows: ET; 230.1 → 186, 103 V/9 eV, ET-d9; 239.1 → 195.1, 98 V/9 eV, Hercynine; 198.1 → 95.1, 94 V/21 eV, Hercynine-d9; 207.2 → 95.1, 97 V/21 eV, S-methyl-ET; 244.1 → 141, 92 V/17 eV, and ET-SO_3_H; 278.1 → 154.1, 120 V/15 eV.

### Whole blood hemoglobin assay

Hemoglobin concentrations in whole blood were determined using the alkaline hematin method. Briefly, whole blood samples were diluted 100 × in Alkaline Hematin Detergent-575 (AHD-575) reagent (2.5% Triton X-100 in 0.1 M NaOH). The absorbance at 575 nm was measured on a Synergy H1 microplate reader (BioTek, VT, USA). A standard curve was generated by dissolving pure hemin (Sigma) in AHD-575 reagent and hemoglobin concentrations were calculated based on the ratio of 4 hemes per hemoglobin molecule.

### Tissue hemoglobin assay

As the alkaline hematin assay is not sensitive enough to determine tissue hemoglobin content, we utilized a heme fluorescence assay adapted from Sinclar *et al*.^63^ to estimate the amount of blood in tissues by measuring the amount of porphyrin. 2–5 mg of tissues were accurately weighed and homogenized in 150 µl of PBS. The samples were centrifuged (5000 *g*, 10 min, 4 °C) before transferring the supernatant into new tubes. Equal volumes of diluted samples were mixed with warmed 2 M oxalic acid and heated at 97 °C for 30 min. Samples were cooled to room temperature before determining the fluorescence (Ex/Em: 407/606 nm) on a Synergy H1 microplate reader. A standard curve was generated by dissolving pure hemoglobin (bovine) in PBS and following the same treatment as the samples. Background fluorescence was accounted for using a set of sample blanks generated by repeating the experiment without heating. The method was also applied to whole blood for a more accurate tabulation of tissue blood content.

### Gene expression analysis on mice liver

All extraction and assay kits were purchased from Qiagen (Hilden, Germany) unless otherwise stated, and all procedures were performed according to the manufacturer’s protocol. RNA from liver samples kept in RNAlater solution was extracted using the RNeasy Mini kit with on-column DNase. Extracted RNAs were quantified and checked by 260/280 nm measurement using the Synergy H1 spectrophotometer with Take 3 microvolume plate (BioTek, VT, USA). Reverse-transcription PCR (RT-PCR) was performed using OneStep RT-PCR kit, and bands resolved on a 2% agarose gel and visualised with GelRed dye (Biotium, CA, USA). Bands were imaged on a Chemidoc (Bio-Rad, CA, USA) and densitometrically quantified using ImageJ software.

### Statistical analysis

Data were tabulated by using Microsoft Excel (Microsoft Corporation, WA, USA). Statistical analyses were done by Graphpad Prism version 6 (Graphpad, CA, USA). Data are expressed as mean – standard error of the mean, with p < 0.05 considered statistically significant. Pearson’s correlation coefficient was used to calculate associations between variables with p < 0.05, indicating significance of the correlation coefficient.

## Electronic supplementary material

Supplementary Data

## References

[1] Tanret, C. Sur une base nouvelle retiree du seigle ergote, l’ergothioneine. *Compt Rend*, 222–224 (1909).

[2] Carlsson J, Kierstan MP, Brocklehurst K (1974). Reactions of L-ergothioneine and some other aminothioneswith2,2′-and 4,4′-dipyridyl disulphides and of L-ergothioneine with iodoacetamide. 2-Mercaptoimidazoles, 2- and 4-thiopyridones, thiourea and thioacetamide as highly reactive neutral sulphur nucleophils. The Biochemical journal.

[3] Bello MH, Barrera-Perez V, Morin D, Epstein L (2012). The Neurospora crassa mutant NcDeltaEgt-1 identifies an ergothioneine biosynthetic gene and demonstrates that ergothioneine enhances conidial survival and protects against peroxide toxicity during conidial germination. Fungal Genet Biol.

[4] Sheridan KJ (2016). Ergothioneine Biosynthesis and Functionality in the Opportunistic Fungal Pathogen, Aspergillus fumigatus. Sci Rep.

[5] Pluskal T, Ueno M, Yanagida M (2014). Genetic and metabolomic dissection of the ergothioneine and selenoneine biosynthetic pathway in the fission yeast, S. pombe, and construction of an overproduction system. PLoS One.

[6] Genghof DS, Inamine E, Kovalenko V, Melville DB (1956). Ergothioneine in microorganisms. The Journal of biological chemistry.

[7] Genghof DS, Vandamme O (1964). Biosynthesis of ergothioneine and hercynine by mycobacteria. Journal of bacteriology.

[8] Narainsamy K (2016). Oxidative-stress detoxification and signalling in cyanobacteria: the crucial glutathione synthesis pathway supports the production of ergothioneine and ophthalmate. Mol Microbiol.

[9] Pfeiffer C, Bauer T, Surek B, Schömig E, Gründemann D (2011). Cyanobacteria produce high levels of ergothioneine. Food Chemistry.

[10] Seebeck FP (2010). *In vitro* reconstitution of Mycobacterial ergothioneine biosynthesis. J Am Chem Soc.

[11] Alamgir KM, Masuda S, Fujitani Y, Fukuda F, Tani A (2015). Production of ergothioneine by Methylobacterium species. Front Microbiol.

[12] Cheah IK, Halliwell B (2012). Ergothioneine; antioxidant potential, physiological function and role in disease. Biochimica et Biophysica Acta (BBA) - Molecular Basis of Disease.

[13] Cheah IK, Tang RM, Yew TS, Lim KH, Halliwell B (2017). Administration of Pure Ergothioneine to Healthy Human Subjects: Uptake, Metabolism, and Effects on Biomarkers of Oxidative Damage and Inflammation. Antioxid Redox Signal.

[14] Grundemann D (2005). Discovery of the ergothioneine transporter. Proceedings of the National Academy of Sciences of the United States of America.

[15] Kato Y (2010). Gene knockout and metabolome analysis of carnitine/organic cation transporter OCTN1. Pharmaceutical research.

[16] Ey J, Schomig E, Taubert D (2007). Dietary sources and antioxidant effects of ergothioneine. Journal of agricultural and food chemistry.

[17] Weigand-Heller AJ, Kris-Etherton PM, Beelman RB (2012). The bioavailability of ergothioneine from mushrooms (Agaricus bisporus) and the acute effects on antioxidant capacity and biomarkers of inflammation. Prev Med.

[18] Kalaras MD, Richie JP, Calcagnotto A, Beelman RB (2017). Mushrooms: A rich source of the antioxidants ergothioneine and glutathione. Food Chem.

[19] Melville DB, Horner WH, Lubschez R (1954). Tissue ergothioneine. The Journal of biological chemistry.

[20] Shires TK, Brummel MC, Pulido JS, Stegink LD (1997). Ergothioneine distribution in *bovine and p*orcine ocular tissues. Comparative biochemistry and physiology Part C, Pharmacology, toxicology & endocrinology.

[21] Salt HB (1931). The ergothioneine content of the blood in health and disease. The Biochemical journal.

[22] Leone E, Mann T (1951). Ergothioneine in the seminal vesicle secretion. Nature.

[23] Halliwell B, Cheah IK, Drum CL (2016). Ergothioneine, an adaptive antioxidant for the protection of injured tissues? A hypothesis. Biochem Biophys Res Commun.

[24] Franzoni F (2006). An in vitro study on the free radical scavenging capacity of ergothioneine: comparison with reduced glutathione, uric acid and trolox. Biomedecine & pharmacotherapie.

[25] Rougee M, Bensasson RV, Land EJ, Pariente R (1988). Deactivation of singlet molecular oxygen by thiols and related compounds, possible protectors against skin photosensitivity. Photochemistry and photobiology.

[26] Colognato R (2006). Modulation of hydrogen peroxide-induced DNA damage, MAPKs activation and cell death in PC12 by ergothioneine. Clin Nutr.

[27] Laurenza I, Colognato R, Migliore L, Del Prato S, Benzi L (2008). Modulation of palmitic acid-induced cell death by ergothioneine: evidence of an anti-inflammatory action. Biofactors.

[28] Rahman I (2003). Ergothioneine inhibits oxidative stress- and TNF-alpha-induced NF-kappa B activation and interleukin-8 release in alveolar epithelial cells. Biochem Biophys Res Commun.

[29] Motohashi N, Mori I, Sugiura Y, Tanaka H (1977). Radioprotective effect of ergothioneine on gamma-irradiation of metmyoglobin: comparison with cysteine on sulfmyoglobin-formation. Chem Pharm Bull (Tokyo).

[30] Hartman PE, Hartman Z, Citardi MJ (1988). Ergothioneine, histidine, and two naturally occurring histidine dipeptides as radioprotectors against gamma-irradiation inactivation of bacteriophages T4 and P22. Radiat Res.

[31] Hseu YC (2015). Dermato-protective properties of ergothioneine through induction of Nrf2/ARE-mediated antioxidant genes in UVA-irradiated Human keratinocytes. Free Radic Biol Med.

[32] Cheah IK (2016). Liver ergothioneine accumulation in a guinea pig model of non-alcoholic fatty liver disease. A possible mechanism of defence?. Free Radic Res.

[33] Moncaster JA, Walsh DT, Gentleman SM, Jen LS, Aruoma OI (2002). Ergothioneine treatment protects neurons against N-methyl-D-aspartate excitotoxicity in an *in vivo* rat retinal model. Neurosci Lett.

[34] Song TY, Chen CL, Liao JW, Ou HC, Tsai MS (2010). Ergothioneine protects against neuronal injury induced by cisplatin both *in vitro* and *in vivo*. Food Chem Toxicol.

[35] Cheah IK, Feng L, Tang RM, Lim KH, Halliwell B (2016). Ergothioneine levels in an elderly population decrease with age and incidence of cognitive decline; a risk factor for neurodegeneration?. Biochem Biophys Res Commun.

[36] Hatano T, Saiki S, Okuzumi A, Mohney RP, Hattori N (2016). Identification of novel biomarkers for Parkinson’s disease by metabolomic technologies. Journal of neurology, neurosurgery, and psychiatry.

[37] Servillo L, D’Onofrio N, Balestrieri ML (2017). Ergothioneine Antioxidant Function: From Chemistry to Cardiovascular Therapeutic Potential. J Cardiovasc Pharmacol.

[38] Marone PA, Trampota J, Weisman S (2016). A Safety Evaluation of a Nature-Identical l-Ergothioneine in Sprague Dawley Rats. Int J Toxicol.

[39] Nakamichi N (2016). Food-derived hydrophilic antioxidant ergothioneine is distributed to the brain and exerts antidepressant effect in mice. Brain Behav.

[40] Servillo L (2015). An uncommon redox behavior sheds light on the cellular antioxidant properties of ergothioneine. Free Radic Biol Med.

[41] Sotgia S (2016). Identification of the Main Intermediate Precursor of l-Ergothioneine Biosynthesis in Human Biological Specimens. Molecules.

[42] Bachmanov AA, Reed DR, Beauchamp GK, Tordoff MG (2002). Food intake, water intake, and drinking spout side preference of 28 mouse strains. Behav Genet.

[43] Mitsuyama H, May JM (1999). Uptake and antioxidant effects of ergothioneine in human erythrocytes. Clin Sci (Lond).

[44] Wang LZ (2013). Quantification of L-ergothioneine in human plasma and erythrocytes by liquid chromatography-tandem mass spectrometry. J Mass Spectrom.

[45] Arduini A, Eddy L, Hochstein P (1990). The reduction of ferryl myoglobin by ergothioneine: a novel function for ergothioneine. Arch Biochem Biophys.

[46] Bedirli A (2004). Ergothioneine pretreatment protects the liver from ischemia-reperfusion injury caused by increasing hepatic heat shock protein 70. J Surg Res.

[47] Spicer SS, Wooley JG, Kessler V (1951). Ergothioneine depletion in rabbit erythrocytes and its effect on methemoglobin formation and reversion. Proc Soc Exp Biol Med.

[48] Kawano H, Higuchi F, Mayumi T, Hama T (1982). Studies on ergothioneine. VII. Some effects on ergothioneine on glycolytic metabolism in red blood cells from rats. Chem Pharm Bull (Tokyo).

[49] Shinozaki Y. *et al*. Impairment of the carnitine/organic cation transporter 1-ergothioneine axis is mediated by intestinal transporter dysfunction in chronic kidney disease. *Kidney Int*, In press (2017). 10.1016/j.kint.2017.04.032 28754554

[50] Santosa S, Jones PJ (2005). Oxidative stress in ocular disease: does lutein play a protective role?. CMAJ.

[51] Kimura A (2017). Targeting Oxidative Stress for Treatment of Glaucoma and Optic Neuritis. Oxid Med Cell Longev.

[52] Abu-Amero KK, Kondkar AA, Chalam KV (2016). Resveratrol and Ophthalmic Diseases. Nutrients.

[53] Kim EC (2017). Screening and Characterization of Drugs That Protect Corneal Endothelial Cells Against Unfolded Protein Response and Oxidative Stress. Invest Ophthalmol Vis Sci.

[54] Wu X (2000). Structural and functional characteristics and tissue distribution pattern of rat OCTN1, an organic cation transporter, cloned from placenta. Biochim Biophys Acta.

[55] Nakamura T, Yoshida K, Yabuuchi H, Maeda T, Tamai I (2008). Functional characterization of ergothioneine transport by rat organic cation/carnitine transporter Octn1 (slc22a4). Biol Pharm Bull.

[56] Jang JH, Aruoma OI, Jen LS, Chung HY, Surh YJ (2004). Ergothioneine rescues PC12 cells from beta-amyloid-induced apoptotic death. Free Radic Biol Med.

[57] Ishimoto T (2014). Organic cation transporter-mediated ergothioneine uptake in mouse neural progenitor cells suppresses proliferation and promotes differentiation into neurons. PLoS One.

[58] Heath H, Rimington C, Searle CE, Lawson A (1952). Some effects of administering ergothioneine to rats. The Biochemical journal.

[59] Heath H (1953). The metabolism of 35S-labelled 2-thiolhistidine and ergothioneine in the rat. The Biochemical journal.

[60] Grigat S (2007). Probing the substrate specificity of the ergothioneine transporter with methimazole, hercynine, and organic cations. Biochem Pharmacol.

[61] Askari A, Melville DB (1962). The reaction sequence in ergothioneine biosynthesis: hercynine as an intermediate. The Journal of biological chemistry.

[62] Redford-Ellis M, Gowenlock AH (1971). Studies on the reaction of chloromethane with human blood. Acta Pharmacol Toxicol (Copenh).

[63] Sinclair, P. R., Gorman, N. & Jacobs, J. M. Measurement of heme concentration. *Curr Protoc Toxico*l Chapter 8, Unit8 3 (2001). 10.1002/0471140856.tx0803s00 20954156

