# Supplementary material for: Distribution and accumulation of dietary ergothioneine and its metabolites in mouse tissues
Source: Sci Rep. 2018 Jan 25;8:1601. doi: 10.1038/s41598-018-20021-z (PMC5785509; doi:10.1038/s41598-018-20021-z)
Supplement: Supplementary file 1 — Supplementary Data [file 41598_2018_20021_MOESM1_ESM.pdf]

Distribution and accumulation of dietary ergothioneine and its metabolites in mouse tissues

(Supplementary Data)

Richard Ming Yi Tang[1], Irwin Kee-Mun Cheah[2], Terry Shze Keong Yew[2], Barry Halliwell*[2]

1 National University of Singapore Graduate School for Integrative Sciences and Engineering
2 Department of Biochemistry, Yong Loo Lin School of Medicine,
National University of Singapore, 28 Medical Drive, Singapore

* To whom all correspondence should be addressed:
Barry Halliwell;
Centre for Life Sciences,
28 Medical Drive, #04-19A, Singapore 117456
Tel.: +65 65163247
Fax: +65 67773271
E-mail address: bchbh@nus.edu.sg

Supplementary Table 1: Statistical analysis of ET uptake in various mouse tissues.

**Kruskal-Wallis test**	**Significance**	**Dunn's Post Hoc**	**Significance**
Whole Blood_1D	ns	-	-
Whole Blood_7D	****	Control vs. ET+	*
		Control vs. ET++	***
		ET+ vs. ET++	ns
Whole Blood_28D	****	Control vs. ET+	**
		Control vs. ET++	**
		ET+ vs. ET++	ns
Liver_1D	*	Control vs. ET+	ns
		Control vs. ET++	*
		ET+ vs. ET++	ns
Liver_7D	***	Control vs. ET+	ns
		Control vs. ET++	***
		ET+ vs. ET++	ns
Liver_28D	***	Control vs. ET+	*
		Control vs. ET++	**
		ET+ vs. ET++	ns
Brain_1D	ns	-	-
Brain_7D	**	Control vs. ET+	ns
		Control vs. ET++	**
		ET+ vs. ET++	ns
Brain_28D	****	Control vs. ET+	*
		Control vs. ET++	***
		ET+ vs. ET++	ns
Eye_1D	ns	-	-
Eye_7D	****	Control vs. ET+	ns
		Control vs. ET++	***
		ET+ vs. ET++	ns
Eye_28D	***	Control vs. ET+	**
		Control vs. ET++	**
		ET+ vs. ET++	ns
Kidney_1D	ns	-	-
Kidney_7D	****	Control vs. ET+	*
		Control vs. ET++	**
		ET+ vs. ET++	ns
Kidney_28D	***	Control vs. ET+	**
		Control vs. ET++	**
		ET+ vs. ET++	ns
Heart_1D	ns	-	-
Heart_7D	****	Control vs. ET+	**
		Control vs. ET++	*
		ET+ vs. ET++	ns
Heart_28D	***	Control vs. ET+	**
		Control vs. ET++	**
		ET+ vs. ET++	ns
Spleen_1D	*	Control vs. ET+	ns
		Control vs. ET++	*
		ET+ vs. ET++	ns
Spleen_7D	*	Control vs. ET+	ns
		Control vs. ET++	*
		ET+ vs. ET++	ns
Spleen_28D	***	Control vs. ET+	*
		Control vs. ET++	**
		ET+ vs. ET++	ns
Lung_1D	ns	-	-
Lung_7D	*	Control vs. ET+	ns
		Control vs. ET++	*
		ET+ vs. ET++	ns
Lung_28D	**	Control vs. ET+	*
		Control vs. ET++	ns
		ET+ vs. ET++	ns
Small Intestine_1D	ns	-	-
Small Intestine_7D	*	Control vs. ET+	ns
		Control vs. ET++	*
		ET+ vs. ET++	ns
Small Intestine_28D	**	Control vs. ET+	*
		Control vs. ET++	*
		ET+ vs. ET++	ns
Large Intestine_1D	*	Control vs. ET+	ns
		Control vs. ET++	*
		ET+ vs. ET++	ns
Large Intestine_7D	***	Control vs. ET+	*
		Control vs. ET++	**
		ET+ vs. ET++	ns
Large Intestine_28D	****	Control vs. ET+	**
		Control vs. ET++	*
		ET+ vs. ET++	ns

ns: not significant p>0.05;
**p<0.05, **p<0.01, ***p<0.001, ****p<0.0001*

Supplementary Table 2: Statistical analysis of frequency-dependent ET uptake in various mouse tissues.

Mann-Whitney Test		Significance
Whole Blood_Control	1D vs. 7D	ns
	7D vs 28D	**
Whole Blood_ET+	1D vs. 7D	***
	7D vs 28D	***
Whole Blood_ET++	1D vs. 7D	***
	7D vs 28D	***
Liver_Control	1D vs. 7D	ns
	7D vs 28D	p = 0.073
Liver_ET+	1D vs. 7D	*
	7D vs 28D	*
Liver_ET++	1D vs. 7D	***
	7D vs 28D	ns
Brain_Control	1D vs. 7D	ns
	7D vs 28D	ns
Brain_ET+	1D vs. 7D	*
	7D vs 28D	***
Brain_ET++	1D vs. 7D	**
	7D vs 28D	**
Eye_Control	1D vs. 7D	ns
	7D vs 28D	p = 0.053
Eye_ET+	1D vs. 7D	***
	7D vs 28D	**
Eye_ET++	1D vs. 7D	***
	7D vs 28D	***
Kidney_Control	1D vs. 7D	ns
	7D vs 28D	**
Kidney_ET+	1D vs. 7D	***
	7D vs 28D	p = 0.053
Kidney_ET++	1D vs. 7D	***
	7D vs 28D	ns
Heart_Control	1D vs. 7D	ns
	7D vs 28D	ns
Heart_ET+	1D vs. 7D	***
	7D vs 28D	*
Heart_ET++	1D vs. 7D	**
	7D vs 28D	p = 0.051
Spleen_Control	1D vs. 7D	ns
	7D vs 28D	ns
Spleen_ET+	1D vs. 7D	**
	7D vs 28D	**
Spleen_ET++	1D vs. 7D	**
	7D vs 28D	*
Lung_Control	1D vs. 7D	ns
	7D vs 28D	ns
Lung_ET+	1D vs. 7D	**
	7D vs 28D	ns
Lung_ET++	1D vs. 7D	***
	7D vs 28D	ns
Small Intestine_Control	1D vs. 7D	ns
	7D vs 28D	ns
Small Intestine_ET+	1D vs. 7D	ns
	7D vs 28D	ns
Small Intestine_ET++	1D vs. 7D	*
	7D vs 28D	ns
Large Intestine_Control	1D vs. 7D	ns
	7D vs 28D	ns
Large Intestine_ET+	1D vs. 7D	**
	7D vs 28D	ns
Large Intestine_ET++	1D vs. 7D	*
	7D vs 28D	ns

ns: not significant p>0.05;
**p<0.05, **p<0.01, ***p<0.001*

Supplementary Table 3: Statistical analysis of dose-dependent ET uptake in various mouse tissues.

**Mann-Whitney Test**		**Significance**
Whole Blood_1D	ET+ vs. ET++	ns
Whole Blood_7D	ET+ vs. ET++	p = 0.073
Whole Blood_28D	ET+ vs. ET++	ns
Liver_1D	ET+ vs. ET++	*
Liver_7D	ET+ vs. ET++	*
Liver_28D	ET+ vs. ET++	ns
Brain_1D	ET+ vs. ET++	ns
Brain_7D	ET+ vs. ET++	ns
Brain_28D	ET+ vs. ET++	p = 0.053
Eye_1D	ET+ vs. ET++	ns
Eye_7D	ET+ vs. ET++	*
Eye_28D	ET+ vs. ET++	ns
Kidney_1D	ET+ vs. ET++	ns
Kidney_7D	ET+ vs. ET++	ns
Kidney_28D	ET+ vs. ET++	ns
Heart_1D	ET+ vs. ET++	ns
Heart_7D	ET+ vs. ET++	ns
Heart_28D	ET+ vs. ET++	ns
Spleen_1D	ET+ vs. ET++	ns
Spleen_7D	ET+ vs. ET++	ns
Spleen_28D	ET+ vs. ET++	ns
Lung_1D	ET+ vs. ET++	ns
Lung_7D	ET+ vs. ET++	p = 0.073
Lung_28D	ET+ vs. ET++	ns
Small Intestine_1D	ET+ vs. ET++	ns
Small Intestine_7D	ET+ vs. ET++	ns
Small Intestine_28D	ET+ vs. ET++	ns
Large Intestine_1D	ET+ vs. ET++	ns
Large Intestine_7D	ET+ vs. ET++	ns
Large Intestine_28D	ET+ vs. ET++	ns

*ns: not significant p>0.05; *p<0.05*

Supplementary Table 4: Liver and brain ET levels after accounting for tissue blood ET

Samples	Days of feeding	ET levels	Total tissue ET (ng/mg)	Tissue ET (minus blood ET) (ng/mg)	% change
Liver50	1	Saline	88.17	87.11	1.2%
Liver6	1	ET+	114.68	114.08	0.5%
Liver10	1	ET++	158.42	157.92	0.3%
Liver58	7	Saline	83.46	82.50	1.2%
Liver26	7	ET+	180.76	179.84	0.5%
Liver17	7	ET++	238.72	237.64	0.5%
Liver45	28	Saline	80.52	79.50	1.3%
Liver30	28	ET+	248.03	246.44	0.6%
Liver34	28	ET++	275.51	273.31	0.8%
Brain10	1	ET++	3.74	3.53	5.5%
Brain17	7	ET++	6.61	6.12	7.4%
Brain34	28	ET++	8.85	8.54	3.4%

Supplementary Data 1: Hemoglobin levels in whole blood. (A) Hemoglobin concentrations for all 63 animals determined by AHD assay. (B) No significant correlation of ET with hemoglobin concentration in whole blood.

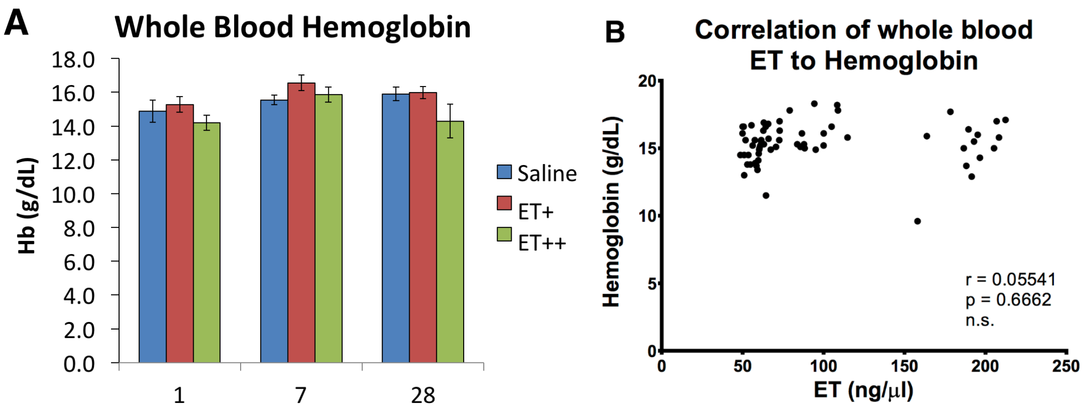

Supplementary Data 2: ET and hercynine levels in tissues after accounting for blood content (n=7). (A-C) Hemoglobin content in all lung, spleen and kidney tissues. (D-I) Lung, spleen, and kidney ET levels were corrected by calculating the amount of blood in the tissues and subtracting the amount of blood ET from the total tissue ET obtained by LC-MS/MS. (J-O) Lung, spleen, and kidney hercynine levels after correcting for blood hercynine contribution. (P) Average percentage changes between total and normalized lung, spleen, and kidney ET and hercynine concentrations.

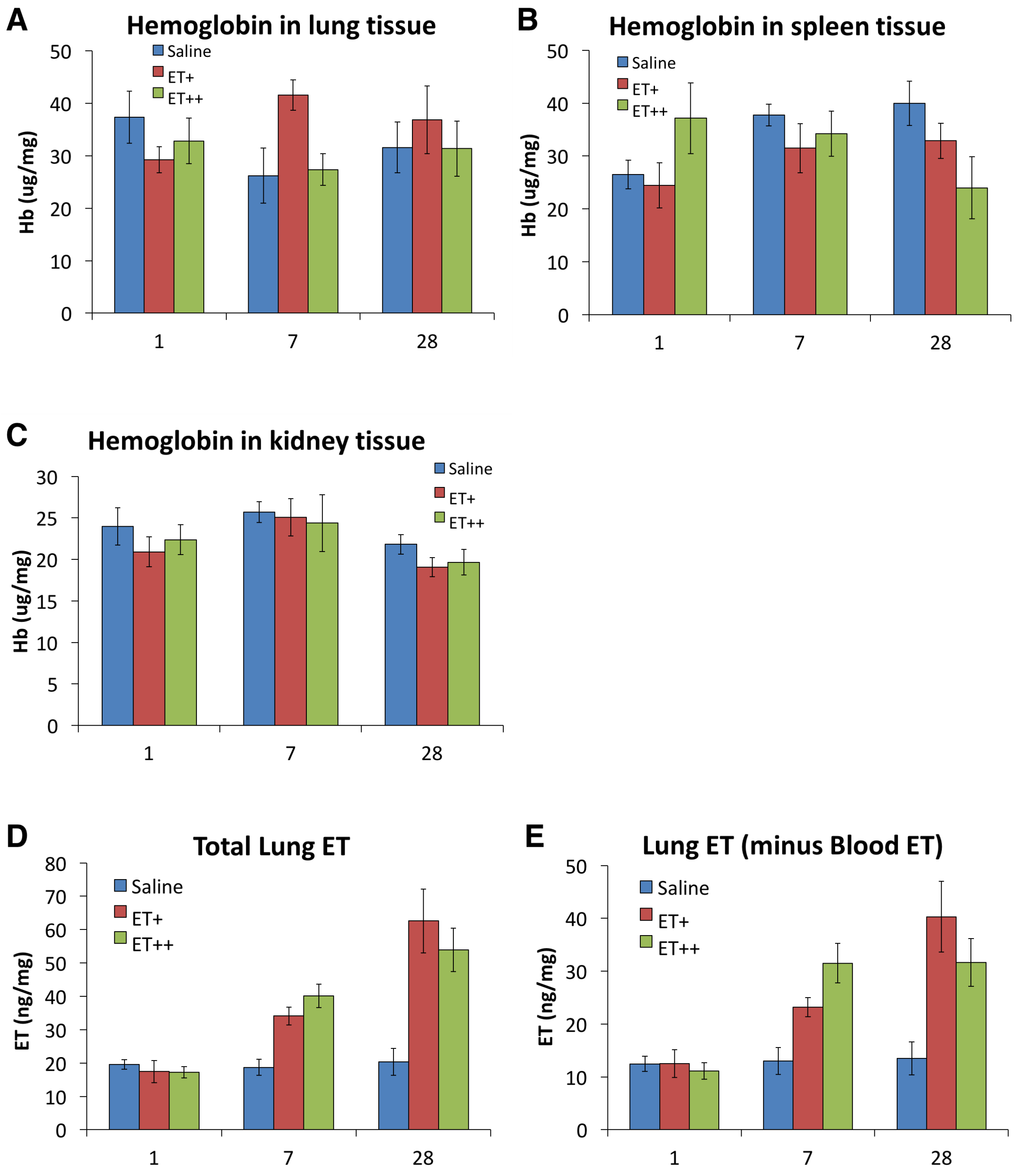

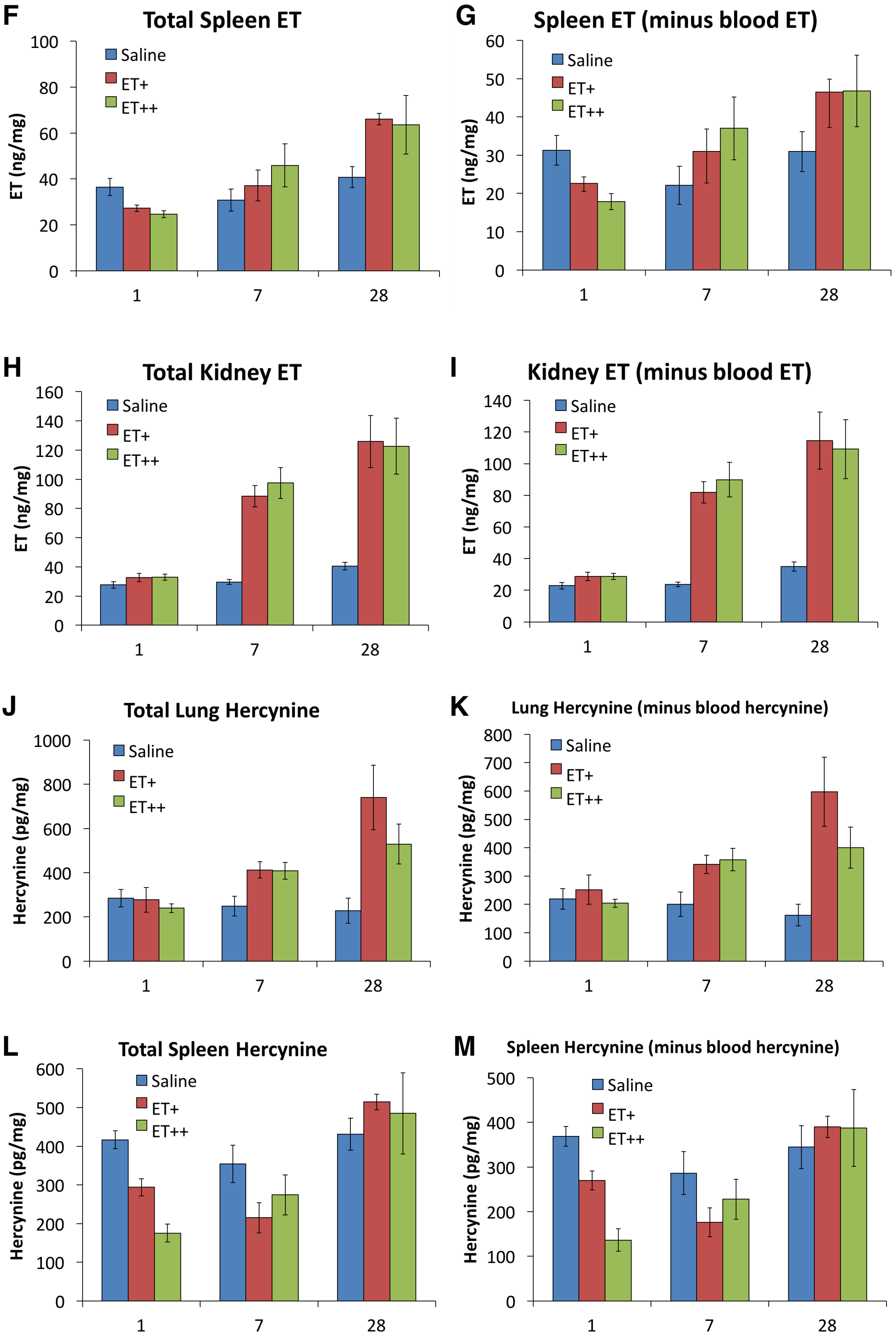
F
Total Spleen ET
ET (ng/mg)
0
20
40
60
80
100
Saline
ET+
ET++
1
7
28
G
Spleen ET (minus blood ET)
ET (ng/mg)
0
10
20
30
40
50
60
Saline
ET+
ET++
1
7
28
H
Total Kidney ET
ET (ng/mg)
0
20
40
60
80
100
120
140
160
Saline
ET+
ET++
1
7
28
I
Kidney ET (minus blood ET)
ET (ng/mg)
0
20
40
60
80
100
120
140
Saline
ET+
ET++
1
7
28
J
Total Lung Hercynine
Hercynine (pg/mg)
0
200
400
600
800
1000
Saline
ET+
ET++
1
7
28
K
Lung Hercynine (minus blood hercynine)
Hercynine (pg/mg)
0
100
200
300
400
500
600
700
800
Saline
ET+
ET++
1
7
28
L
Total Spleen Hercynine
Hercynine (pg/mg)
0
100
200
300
400
500
600
Saline
ET+
ET++
1
7
28
M
Spleen Hercynine (minus blood hercynine)
Hercynine (pg/mg)
0
100
200
300
400
500
Saline
ET+
ET++
1
7
28

N

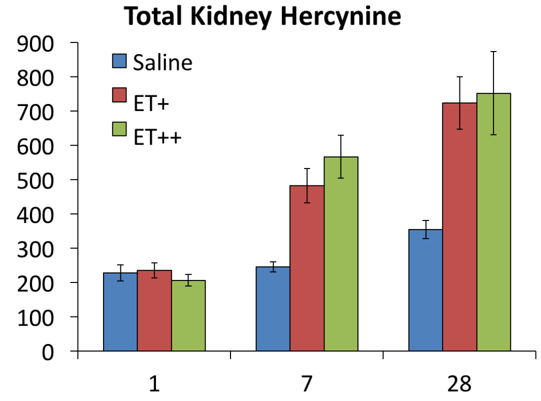

O

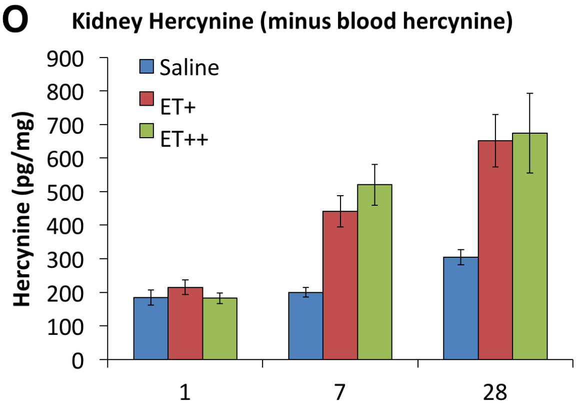

P

Tissues	Percentage change in tissue ET concentration	Percentage change in tissue hercynine
Lung	33.2% ± 12.2%	19.4% ± 9.4%
Spleen	23.8% ± 11.5%	18.8% ± 9.3%
Kidney	12.7% ± 5.4%	12.7% ± 5.5%

Supplementary Data 3: Correlation data between ET and hercynine, ET-SO_3H and S-methyl ET, and whole blood ET with tissue ET.

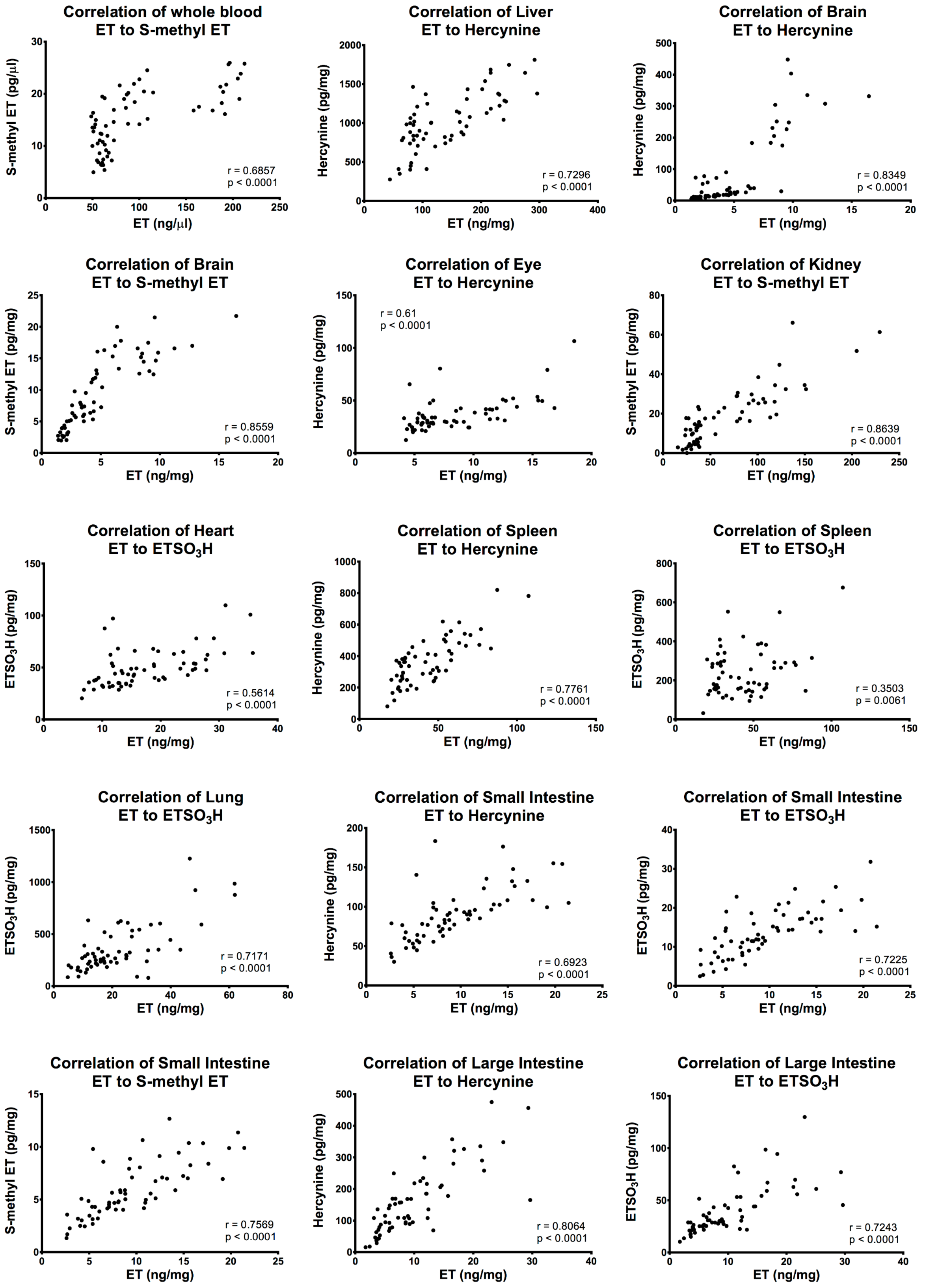

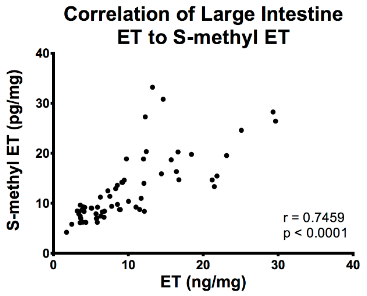
Correlation of Large Intestine ET to S-methyl ET
S-methyl ET (pg/mg)
ET (ng/mg)
r = 0.7459
p < 0.0001

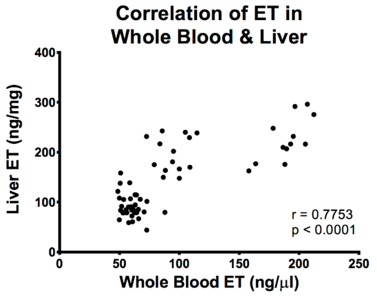
Correlation of ET in Whole Blood & Liver
Liver ET (ng/mg)
Whole Blood ET (ng/μl)
r = 0.7753
p < 0.0001

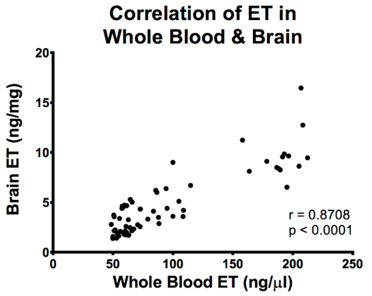
Correlation of ET in Whole Blood & Brain
Brain ET (ng/mg)
Whole Blood ET (ng/μl)
r = 0.8708
p < 0.0001

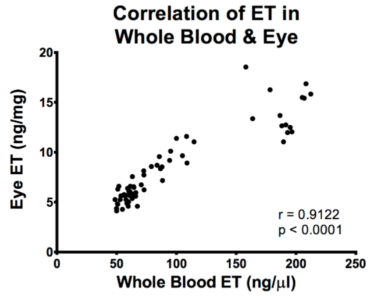
Correlation of ET in Whole Blood & Eye
Eye ET (ng/mg)
Whole Blood ET (ng/μl)
r = 0.9122
p < 0.0001

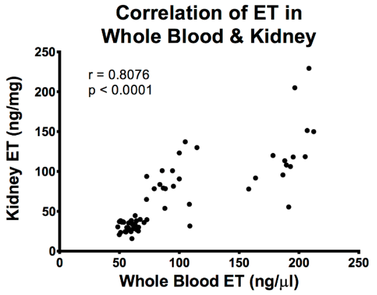
Correlation of ET in Whole Blood & Kidney
Kidney ET (ng/mg)
Whole Blood ET (ng/μl)
r = 0.8076
p < 0.0001

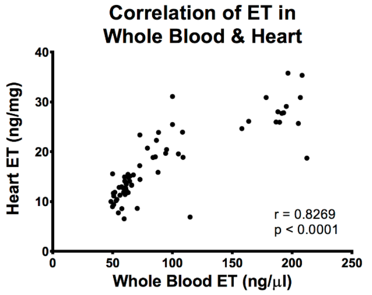
Correlation of ET in Whole Blood & Heart
Heart ET (ng/mg)
Whole Blood ET (ng/μl)
r = 0.8269
p < 0.0001

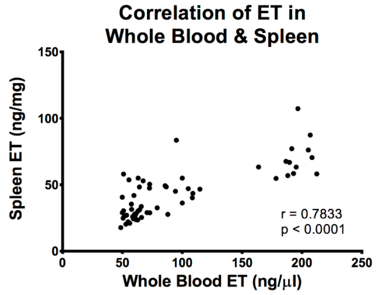
Correlation of ET in Whole Blood & Spleen
Spleen ET (ng/mg)
Whole Blood ET (ng/μl)
r = 0.7833
p < 0.0001

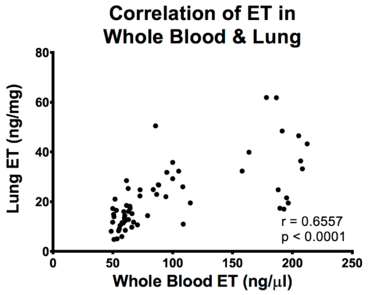
Correlation of ET in Whole Blood & Lung
Lung ET (ng/mg)
Whole Blood ET (ng/μl)
r = 0.6557
p < 0.0001

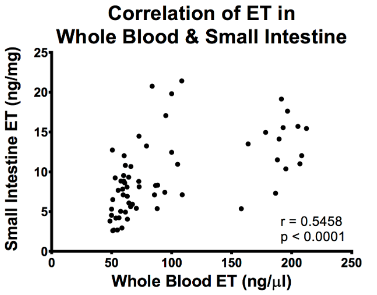
Correlation of ET in Whole Blood & Small Intestine
Small Intestine ET (ng/mg)
Whole Blood ET (ng/μl)
r = 0.5458
p < 0.0001

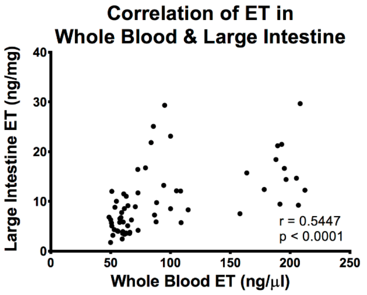
Correlation of ET in Whole Blood & Large Intestine
Large Intestine ET (ng/mg)
Whole Blood ET (ng/μl)
r = 0.5447
p < 0.0001
